# Tumor intrinsic properties dictate Fc receptor expression and cancer cachexia associated increase in checkpoint inhibitor clearance

**DOI:** 10.3389/fimmu.2025.1669979

**Published:** 2025-12-17

**Authors:** Bryan C. Remaily, Greg Young, Hannah Lathrop, Justin Thomas, Kyeongmin Kim, Trang T. Vu, Adeoluwa Adeluola, Camille Stanton, Gillian Mulcahy, Zhiliang Xie, Lauren Granchie, Yizhen Guo, Min Hai, Jessica Wedig, Maria Schmidt, Millennium Manna, Xiaokui Mo, Jeovanna Lowe, Jill A. Rafael-Fortney, Edmund Folefac, Paul Gregorevic, Dwight H. Owen, Samuel K. Kulp, Latha P. Ganesan, Christopher C. Coss, Thomas A. Mace, Mitch A. Phelps

**Affiliations:** 1 Division of Pharmaceutics and Pharmacology, College of Pharmacy, The Ohio State University, Columbus, OH, United States; 2 James Comprehensive Cancer Center, The Ohio State University, Columbus, OH, United States; 3 Department of Biomedical Informatics, College of Medicine, The Ohio State University, Columbus, OH, United States; 4 The Pelotonia Institute for Immuno-Oncology, The Ohio State University, Columbus, OH, United States; 5 Center for Biostatistics, College of Medicine, The Ohio State University, Columbus, OH, United States; 6 Department of Physiology and Cell Biology, College of Medicine, The Ohio State University, Columbus, OH, United States; 7 Davis Heart and Lung Research Institute, College of Medicine, The Ohio State University, Columbus, OH, United States; 8 Division of Medical Oncology, The Ohio State University James Comprehensive Cancer Center, Columbus, OH, United States; 9 Centre for Muscle Research, Department of Anatomy and Physiology, Faculty of Medicine, Dentistry and Health Sciences, The University of Melbourne, Parkville, VIC, Australia; 10 Department of Neurology, University of Washington School of Medicine, Seattle, WA, United States; 11 Department of Internal Medicine, The Ohio State University, Columbus, OH, United States; 12 Division of Gastroenterology, Hepatology & Nutrition, Department of Medicine, The Ohio State University, Columbus, OH, United States

**Keywords:** immune checkpoint inhibitor, antibody pharmacology, cancer cachexia, Fc receptors, FcRn, Fc gamma receptors, antibody catabolism

## Abstract

**Purpose:**

Patients with cancer cachexia display a general resistance to immune checkpoint inhibitor (ICI) therapy, and baseline ICI catabolic clearance is a predictive indicator for overall survival, independent of dose and drug exposure. Fc-gamma (FcγRs) and neonatal Fc receptors (FcRn) play key roles in ICI clearance and efficacy, and we aimed to determine the impact of cachexia, independent of tumor, on immune cell populations and their Fc receptor (FcR) expression in patients and in murine models of cancer, cachexia, and cancer cachexia.

**Experimental design:**

Immune cell populations and their FcR expression were measured in tumor-bearing and tumor-free mice, with/without cachexia, and from patients with non-small cell lung cancer (NSCLC) and renal cell carcinoma. These measures, upon splenocytes and peripheral blood mononuclear cells (PBMCs) in mice and humans respectively, were compared with baseline ICI drug clearance and cachexia phenotype.

**Results:**

Leukocyte populations and FcγR in mouse splenocytes displayed distinct expressional patterns when comparing across tumor and cachexia status. Univariate analyses revealed several correlations between FcγR expression on patient PBMCs and both ICI clearance and cachexia phenotype. Notably, FcRn expression was unchanged or slightly elevated in tumor-bearing mice and did not correlate with ICI clearance in murine splenocytes or patient leukocytes. Furthermore, immune cell populations and FcR expression were different among tumor types but did not differ in splenocytes of tumor-free mice with Activin A/IL-6 induced cachexia when compared with vector controls.

**Conclusions:**

These findings provide the first evidence that FcRs, critical for the efficacy and pharmacokinetics of many ICI and other IgG mAbs, are altered in a tumor-dependent manner. Furthermore, in the absence of a tumor, cachexia phenotype may not coincide with inflammation in the form of altered immune cell populations and elevated catabolic clearance of IgG mAbs, suggesting these features arise from properties intrinsic to the tumor.

## 1 Introduction

Immune checkpoint inhibitors (ICIs) are monoclonal antibody (mAb) drugs that stimulate anti-tumor immune responses and have drastically altered the treatment regimens of solid malignancies over the past decade (1). Anti-PD-1 ICIs such as pembrolizumab have improved progression free survival and overall survival compared to chemotherapy in non-small cell lung cancer (NSCLC), renal cell carcinoma (RCC), microsatellite instability colon cancer, colorectal cancer, and other cancers, without the traditional toxicities associated with chemotherapy (2–5). While promising, only one third of patients receiving ICI therapy achieve durable responses with significant variability across disease types and stages (6, 7).

ICI drug clearance (CL), both at baseline (CL_0_) and the extent to which CL changes over time, has been demonstrated as a strong prognostic indicator for progression free and overall survival (OS) (3, 8). A seminal retrospective analysis of pembrolizumab in patients with melanoma (KEYNOTE-002) and NSCLC (KEYNOTE-010) found that patients with rapid clearance (4^th^ quartile) had worse OS compared to slower clearance (1^st^ quartile) (8.4 vs. 23.4 months). This effect was independent of dose (2 mg/kg, 10 mg/kg, and 200 mg) and subsequent drug exposure, demonstrating CL_0_ is a prognostics biomarker for the poor clinical outcomes. In this study, patients displaying rapid CL and short OS also displayed clinical features of cancer associated cachexia, including weight loss and hypoalbuminemia (3). Cachexia is a multi-factorial syndrome characterized by irreversible loss of skeletal muscle with or without loss of adipose tissue due to a perpetual, hyper-metabolic inflammatory state, and it is a common feature in cancer patients (9, 10). Though cachexia is often undiagnosed, patients with cancer cachexia diagnoses, or those who display phenotypic features of cancer cachexia, tend to respond less favorably, or have shorter duration of response to ICI therapies (8, 11–14). The mechanisms linking cancer cachexia, increased ICI CL, and refractory disease are not understood and have become the focus of translational research efforts in the field (3, 15).

Our group has investigated CL_0_ of pembrolizumab in Lewis Lung Carcinoma (LLC) and Colon-26 carcinoma (C26) murine models of cancer cachexia, as well as the mild cachexia model, CMT-167, and the non-cachectic MC38 models (16–18). Compared to tumor-free (TF) controls, the three models of cachexia illustrated increased clearance of pembrolizumab, though pembrolizumab clearance was unaffected in the non-cachectic MC38 model, suggesting increased pembrolizumab clearance is not due to tumor status alone (17). Importantly, pembrolizumab targets human PD-1 and does not bind murine PD-1, suggesting increased CL_0_ in the LLC, C26, and CMT-167 models is not attributable to variable region target binding, but may instead implicate the antibody Fc domain and its interaction with Fc receptors (16).

Fc receptors (FcR) are responsible for binding of the Fc constant region of IgG and comprise the neonatal Fc receptor (FcRn) and the Fc-gamma family of receptors (FcγRs). FcRn is expressed throughout the body, and noncompetitive binding of FcRn to IgG and albumin recycles and protects them from their proteolytic lysosomal degradation. FcRn function is a key mediator of IgG and albumin homeostasis, and modulation of FcRn affinity/expression can have profound effects on IgG clearance and half-life (19–21). FcRn has also been implicated in antigen presentation, and it may therefore play a more direct role in anti-tumor immune response (22).

The FcγRs are primarily expressed on leukocytes and are known to impact efficacy of immunotherapies, as Fc: FcγR binding can trigger effector functions such as endocytosis, phagocytosis, cellular cytotoxicity, and cellular activation (23). Recent evidence also demonstrates FcγRs role in mAb pharmacokinetics (PK) (23–27). FcγRs are distinctly characterized as either activating (murine, mFcγRI, III, IV and human, hFcγRI, IIa, III) or inhibitory (mFcγRIIb and hFcγRIIb) (23, 28, 29). Binding to activating and inhibitory receptors will lead to concurrent transduction of activating and inhibitory pathways, respectively, with effector functions depending on the sum of signaling relative to an innate threshold of activation. Predisposition in IgG backbones’ activating to inhibitory binding profiles can drive anti-tumor outcomes in anti-PD-1 and anti-PD-L1 treatments (25, 30). Pembrolizumab is built on a humanized IgG4 backbone intended to minimize FcγR interaction, as IgG4 antibodies have low, but non-negligible, affinity for both human and murine FcγRs (31).

Despite evidence of FcR importance in immunotherapy, there remains a lack of understanding how expression of FcRs may be impacted by cancer presence, associated chronic inflammation, and cancer cachexia (9). With a major role for FcR in the efficacy and CL of ICI therapies, this study sought to investigate how FcR expression and FcR-expressing immune cell populations are altered as a function of tumor presence and cachexia status, and how these alterations may link to IgG mAb PK and outcomes from therapy.

## 2 Methods

### 2.1 Cell culture and *in vivo* tumor studies

Methods for LLC, CMT-167 and MC38 cell culture and *in vivo* tumor studies were described previously (16–18). Cells were confirmed negative for mycoplasma using the Plasmotest kit (Invivogen, San Diego, CA) and grown at 37 °C in a humidified chamber with 5% CO_2_ in Dulbecco’s Modified Eagle Medium (Invitrogen, Waltham, MA) supplemented with 10% fetal bovine serum (Biowest, Riverside, MO) and 1% penicillin-streptomycin. Cells were harvested for injection into mice by trypsinization, subsequently pelleted in growth medium, then resuspended in sterile phosphate buffer saline (PBS) to achieve a concentration of 10.0 x 10^6^ cells/ml for LLC and CMT-167, and 5.0 x 10^6^ cells/ml for MC38.

*In vivo* models of cachexia were completed as previously described (16–18). 8–10 week old C57BL/6J mice (Jackson Laboratories, Bar Harbor, ME) were group-housed under constant photoperiod conditions (12-hour light/12-hour dark), and acclimated for a minimum of 3 days after arrival with *ad libitum* access to water and standard diet for the study duration. All mice were male, as male mice generally develop a more severe cachexia phenotype (32, 33). Body weights were measured and recorded once per week. Mice were randomly assigned to groups of tumor-free (TF) or MC38, LLC, or CMT-167 tumor-bearing (TB). On day 0, mice were injected intramuscularly with LLC or CMT-167 cells (0.5 x 10^6^ cells in 0.05ml) in the right hind limb or subcutaneous with MC38 cells (0.5 x 10^6^ cells in 0.1 ml) in the right flank, and TF mice received injections of PBS. Serial tumor measurements and conversions to mass estimates were performed as previously described (16–18). Terminal tumor-adjusted body weights were calculated by subtracting the measured weight of resected tumor at euthanasia from final body weight at end of study (EOS), and tumor burden was calculated as the terminal tumor mass divided by body weight at EOS. For CO_2_ euthanasia, the CO_2_ flow rate is set to ensure a 30%-70% displacement of the chamber volume/min.

### 2.2 Adeno-associated virus studies

The adeno-associated virus (AAV) model was conducted as described previously (34). 20-week-old mice (Jackson Laboratories, Bar Harbor, ME) were given intramuscular injections of viral vector encoding for the production of IL-6 and Activin-A (Act+IL6; n=8) or control vector (Control; n=8). Nine (9) weeks post injection, the study was terminated and mice were sacrificed and cachexia endpoints were assessed as described previously (17). Terminal plasma levels of IL-6 (Biolegend, Cat #431304) and Activin-A (RnD Systems, Cat# DAC00B) were measured using ELISA according to manufacturer’s instructions.

### 2.3 Pharmacokinetic studies

For pembrolizumab PK studies, a single, intravenous injection of 100ug of pembrolizumab (Sellekchem, Houston, TX, USA) was given at day 14 for tumor studies and at 8 weeks for AAV studies as previously described (16, 18, 35). Afterwards, serial plasma timepoints were taken at 1, 48, 96, 144, 168, and 192 hours post injection. Free pembrolizumab was measured by ELISA (16, 18, 35).

### 2.4 Gastrocnemius cross sectional area analysis

Myofiber areas from mouse groups were measured as described previously (18, 36). Left gastrocnemius muscles from randomly selected mice were resected, mounted in 7% tragacanth (Sigma-Aldrich, St. Louis, MO), and then frozen in liquid nitrogen-cooled isopentane (Sigma-Aldrich). Muscle cross sections were cut to 8μm thickness and stained with rat anti-mouse Laminin-2 primary antibody (1:500 ratio, Sigma-Aldrich) and Alexa-594 conjugated anti-rat secondary antibody (Invitrogen). Cross sections were imaged, and muscle myofibers were semi-automatically measured using Imaris software under blinded conditions (Oxford Instruments, Abingdon, UK).

### 2.5 Splenocyte isolation

Detailed methods have been described previously (37). Upon termination of studies, randomly selected spleens were harvested, placed in PBS on ice, then mechanically separated under aseptic conditions and strained through a 70 µm cell strainer (Fisher Scientific, Waltham, MA, USA). The cells were then centrifuged at 1700 rpm for 5 min of which the supernatant was then aspirated. Splenocytes were resuspended in 10mL red blood cell lysis buffer for 5 min. Afterwards the remaining cells were centrifuged, aspirated, and resuspended in cell freezing media (90% FBS + 10% DMSO) prior to freezing and storing at -80 C until analysis.

### 2.6 Human subjects

Eleven (11) patients with either NSCLC or renal cell carcinoma (RCC) receiving standard of care ICI were enrolled in an IRB-approved, non-interventional study (OSU-20001, IRB#2020C0048). This study protocol followed the Declaration of Helsinki and International Conference on Harmonization Good Clinical Practice (ICH-GCP) guidelines. Written informed consent was obtained from all patients. Patients received pembrolizumab (with or without chemotherapy) or nivolumab and ipilimumab (for RCC) as standard of care per their treating oncologist. Blood serum samples were collected pre-dose and 30 minutes post dose of ICI infusion during the first 4 cycles of treatment for measurement of ICIs and pharmacokinetic analyses.

### 2.7 Cytometry time of flight

For analysis of murine splenocytes, cells were flash thawed in 37°C bead bath, spun down, and resuspended in PBS. 3.0 x 10^6^ cells were then aliquoted, and surface stained using Maxpar SP/LN mouse phenotyping kit (StandardBioTools, San Francisco, CA, USA) according to manufacturer’s protocols with antibodies indicated for surface staining in **Supplementary Table S1**. Human PBMC isolation was performed using density gradient centrifugation on patient whole blood via Ficoll-Pacque (Amersham, Pharmacia Biotech, Bjork-gatan, Sweden), and CyTOF analysis was conducted on baseline PBMC samples as previously reported (37). 500 µl of fresh whole blood was transferred into a sterile microcentrifuge tube, and 5µl of Human TruStain FcX (Biolegend) was added and allowed to incubate for 10 min. Whole blood was then transferred to Maxpar Direct Immune Profiling Assay tubes (Standard BioTools) and gently vortexed to ensure all lyophilized pellet dissolved. Additional metal-labeled antibodies used to stain surface proteins are referenced in **Supplementary Table S2**. One day before analyses, cells were thawed and stained intracellularly with antibodies for FcRn (Clone: 937508, RnD Systems) and Ki-67 (Clone: C63D9, StandardBiotools). Cells were then stained with intercalation solution (Cell-ID Intercalator-Ir, StandardBiotools) diluted to 125 nM in MaxPar Fix and Perm (StandardBiotools). After staining, samples were washed twice with Maxpar cell staining buffer, followed by two washes with EDTA diluted to 5uM in deionized water. Patient peripheral blood mononuclear cells (PBMCs) were transferred to filter cap flow tubes, and Maxpar acquisition solution (StandardBiotools) and EQ Four Element Calibration beads (StandardBiotools) were added to the samples prior to running through the Helios Mass Cytometer, after which the data was analyzed using Cytobank.

### 2.8 Human L3 scan analysis

Computed tomography (CT) axial scans of patients enrolled on OSU-20001 were used for body composition analysis as described previously (38, 39). Patient L3 vertebrae was identified in baseline axial CT image scans then analyzed for body composition using Slice-O-Matic v4.31 (Tomovision, Magog, Canada) by measuring surface area of skeletal muscle (-29 to +150 HU), intramuscular adipose tissue (-190 to -30 HU), visceral adipose tissue (-150 to -50 HU), and subcutaneous adipose tissue (-190 to -30 HU). Skeletal muscle index (SMI) is skeletal muscle surface area normalized by patient height in m^2^. Lean mass index (LMI) was derived by taking validated sex-specific SMI cutoffs (39 and 55 cm^2^/m^2^ for females and males respectively) and dividing by patient SMI (40, 41) to calculate LMI. An LMI ≥ 1 indicates low lean mass, and LMI <1 indicated increasing lean mass.

### 2.9 Pharmacokinetic analyses

Unbound pembrolizumab and nivolumab from clinical samples were measured by ELISA (16–18, 42). Posthoc PK parameters were estimated from the plasma PK data from 34 patients receiving either pembrolizumab (n=20) or nivolumab (n=14) using published nonlinear mixed effects models and first-order conditional estimation method with interaction in NONMEM, Version 7.3 (43, 44). Similarly, murine pembrolizumab concentrations were fit to a linear, intravenous, two compartment models, as described previously (16, 18, 35) to derive individual pharmacokinetic parameter estimations.

### 2.10 Statistics

The cytokine and CyTOF measures were log2 transformed to meet data normality assumptions. Mass of bodies and organs were analyzed using original scale. Analysis of variance (ANOVA) were performed followed by comparisons between groups. Data normality assumptions were confirmed using residual plots of models. For comparison of myofiber fiber cross sectional area (CSA), Vargha-Delaney A-statistics of the muscle fiber CSAs between TB and TF muice was calculated and the difference was evaluated using the Brunner-Munzel test (45). Association analysis was conducted in R Studio v4.3.2 (R Core Team, Vienna, Austria) to obtain e Spearman’s rank correlation coefficients. Correlation analyses were performed separately for each pair of variable and marker. To account for multiple hypothesis testing, p values of Spearman’s rank correlation coefficients were adjusted by Benjamini-Hochberg procedure. All other analyses were conducted in SAS 9.4 (SAS Institute, Cary, NC).

## 3 Results

### 3.1 Phenotypic differences of cachexia in MC38, LLC and CMT-167 tumor-bearing mice

**Figure 1:**
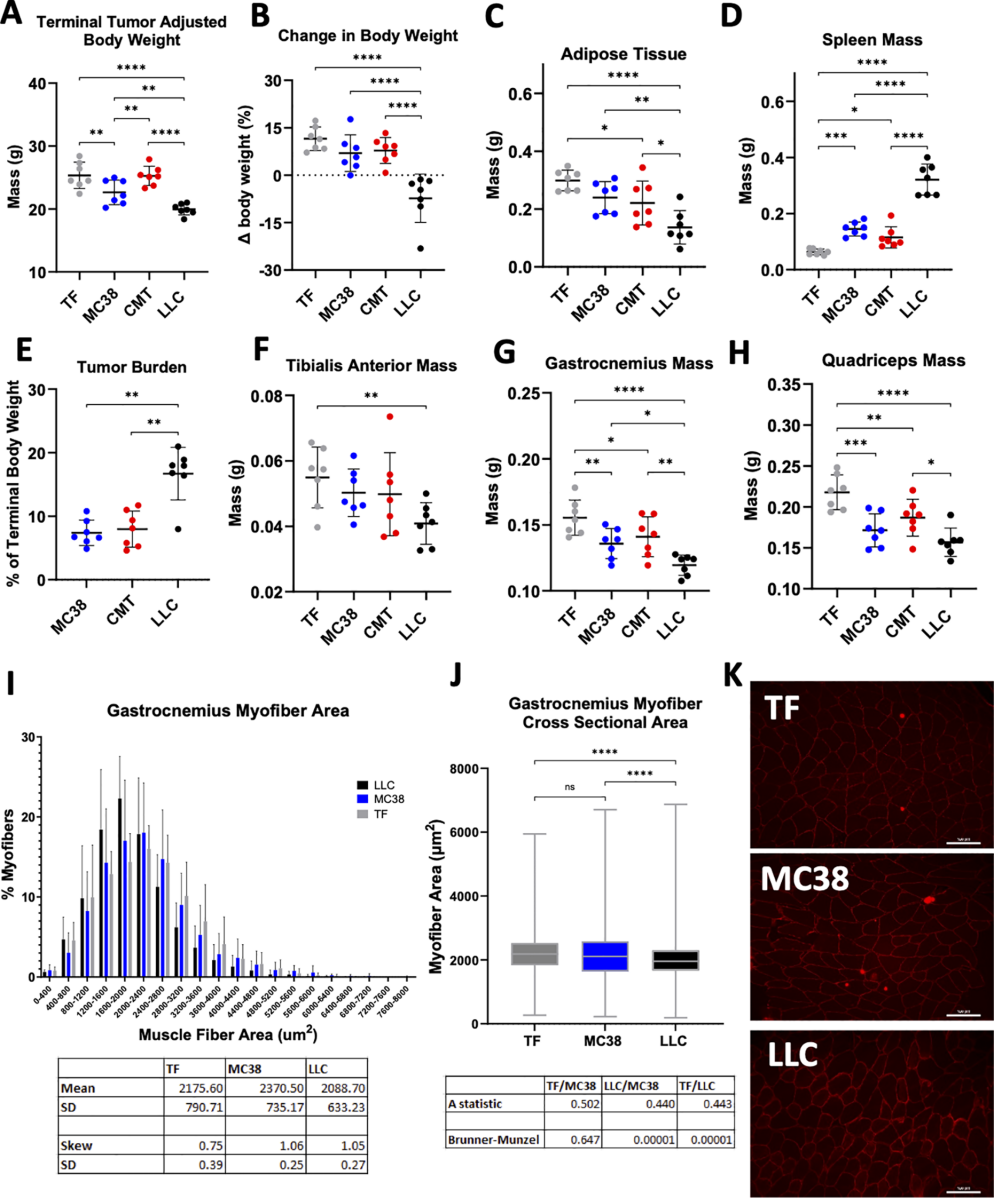
Skeletal muscle atrophy present in LLC mice. Mice that are either TF, or TB with MC38, CMT-167 or LLC tumors (n=7 per group) were euthanized at end of study (EOS) for comparison of tumor-induced effects on body composition and skeletal muscle. At study endpoint **(A)** Tumor-adjusted terminal body weight, total mouse weight at EOS minus the observed tumor mass **(B)** Tumor-adjusted terminal body weight expressed as a percentage of initial (day-1) body weight **(C)** Epididymal adipose tissue mass **(D)** Spleen mass **(E)** Terminal tumor burden, tumor mass as a % of body weight at sacrifice (Mann-Whitney Test) **(F)** Tibialis Anterior (TA) mass **(G)** Gastrocnemius mass and **(H)** Quadriceps mass by ANOVA. **(I)** Gastrocnemius myofiber cross sectional area measurements between TF (n=5) MC38 (n=5) and LLC (n=5) mice. Muscle myofiber area (um^2^) distribution plotted as % of total myofibers (±SD) for individual mice binned every 400 um. Mean SD. **(J)** Gastrocnemius myofiber cross sectional areas plotted by group (±Min/Max). ****P<0.0001 by Brunner-Munzel Test. **(K)** Representative 20x objective images of anti Laminin-2 staining of gastrocnemius myofibers from a healthy C57BL/6J TF mouse and mice with either MC38 or LLC tumors. Mean±SD; *P<0.05; **P<0.01; ***P <0.001; ****P<0.0001.

We previously demonstrated the established murine LLC and CMT-167 cancer cachexia models with severe and mild cachexia phenotypes, respectively, exhibit increased mAb CL (16, 18), and the MC38 tumor model displays greatly reduced cachectic burden without increased mAb CL (16, 35). In the current study, we generally observed expected trends between these three models in tumor adjusted body weight, adipose tissue, and spleen mass (**Figures 1A–D**). Tumor masses in this study were as expected for MC38 and CMT-167 (~8%-10% of terminal body weight), though LLC tumors were larger in the current study (mean 17% of terminal body weight) than in our previous reports (16–18) (**Figure 1E**). Nonetheless, we observed expected trends in muscle tissue mass where a more pronounced loss of mass in tibialis anterior, gastrocnemius, and quadriceps were observed for LLC TB mice compared to either CMT-167 or MC38 TB mice (**Figures 1F–H**). Measurement of myofiber CSA has been demonstrated to correlate with muscle strength and function and enables more rigorous quantification of changes in skeletal muscle morphology and atrophy beyond comparison of simple muscle mass (45, 46). We recently reported gastrocnemius muscle CSA for CMT-167 TB mice (18), and in this current study we show this data for MC38 and LLC TB vs TF mice (**Figures 1I–K**). When comparing TF and MC38, there was no observed change in CSA myofiber distribution or stochastic inequality as determined by Brunner-Munzel test and Vargha-Delaney A statistic, respectively (45). However, myofibers from LLC mice had significantly decreased CSA compared to TF and tumor bearing MC38 groups (**Figures 1J, K**), as we saw previously with CMT-167 TB mice (18). This further demonstrates skeletal muscle losses present in mice bearing CMT-167 and LLC, but not MC38 tumors, indicating skeletal muscle atrophy is not solely a function of tumor presence. Though our tumor models resulted in mixed effects on traditional cachectic outcomes, collectively, our data support MC38, CMT-167, and LLC tumor bearing mice as representative of non-cachectic, mildly cachectic, and severely cachectic models, respectively.

### 3.2 Increased monoclonal antibody clearance in cachectic mice

**Figure 2:**
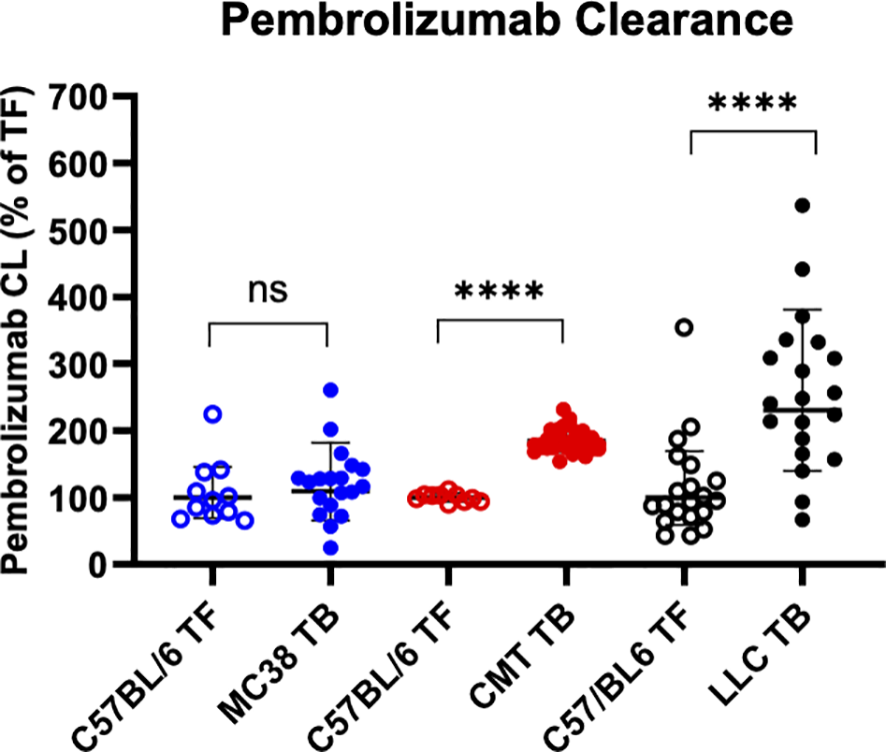
Increased mAb ICI CL in mice with tumor-induced cachexia. Comparison of single dose pembrolizumab CL across tumor models. From left to right: MC38, CMT-167, and LLC mouse CL estimates expressed as a percentage of each groups respective in study tumor free control (mean ± SD). CL for all C57BL/6/LLC mice combined determined by NONMEM analyses as described in Materials and Methods section. *P <0.05; **P<0.01; ***P <0.001; ****P < 0.0001 by Wilcoxin Sign-Rank test.

As we previously demonstrated, the general phenotypic trends in body composition for MC38, CMT-167, and LLC TB mice generally coincide with differences in mAb clearance, with the non-cachectic/mildly cachectic MC-38 TB mice displaying a non-significant difference in mAb clearance compared to TF mice, and both the mildly cachectic CMT-167 and severely cachectic LLC TB mice display significantly elevated mAb clearance (16–18). **Figure 2** displays model-estimated pembrolizumab clearance values for each model versus their respective, within-study tumor free control groups, which demonstrate the differences in mAb clearance in C57BL/6 TF vs TB mice that do (CMT-167 and LLC) or do not (MC38) elicit cachexia.

### 3.3 Circulating immune cell populations differ with respect to tumor status and cachexia phenotype in mice

**Figure 3:**
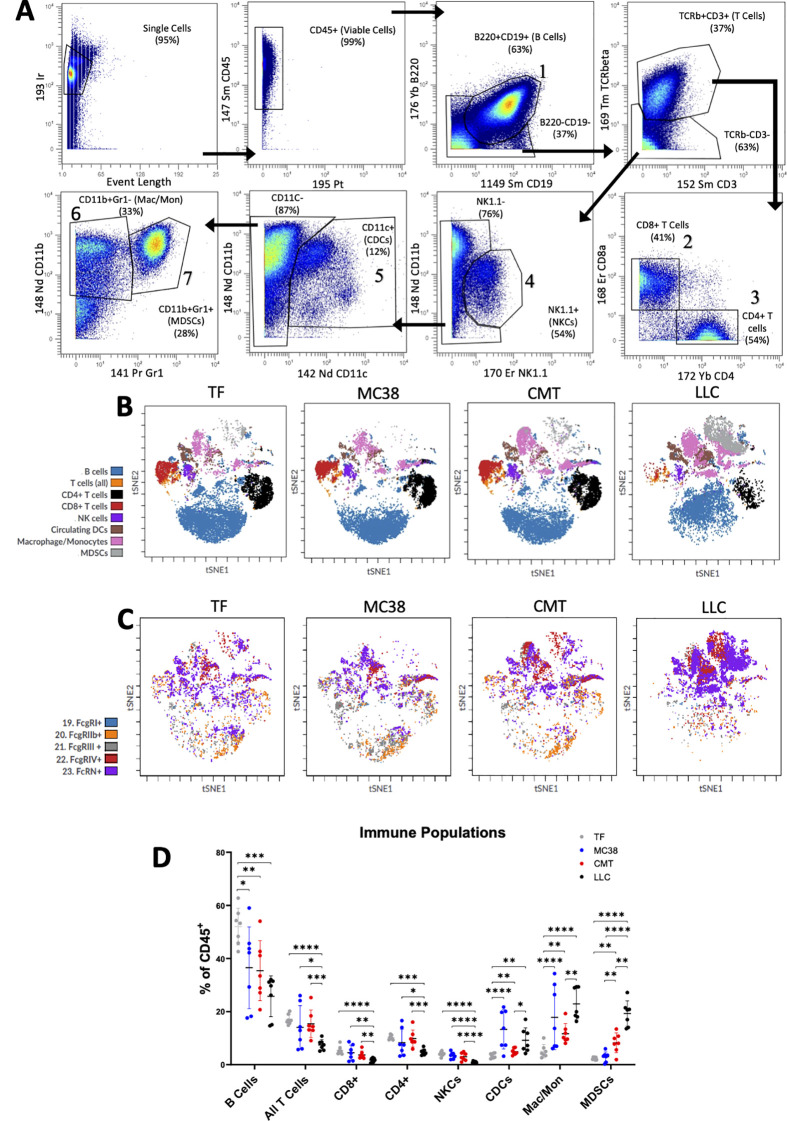
Circulating leukocyte populations altered in pre clinical tumor models. Splenocytes from TF, MC38, CMT-167 and LLC (n=7 per group) mice were stained and measured by CyTOF for analysis of immune populations and FcR expression. **(A)** Representative gating plots for each of the major immune cell populations reported for mice. T-distributed stochastic neighbor embedding (t-SNE) plots showing **(B)** major immune populations and **(C)** immune population Fe Receptor expression. **(D)** Comparisons of overall levels of immune populations expressed as a percent of total immune cells (CD45^+^) across groups. Mean ± SD. *P < 0.05; **P<0.01; ***P<0.001; ****P <0.0001 by ANOVA.

Splenocytes from untreated animals (i.e. the same animals as described in **Figure 1**) were analyzed by CyTOF to understand the relationship between cachexia phenotype, immune cell populations, and FcR expression profiles. Our CyTOF panel comprised 26 markers enabling gating for 17 distinct immune subpopulations plus FcRn and mFcγR (RI, RIIb, RIII, RIV). We chose to report on the major immune populations of B cells, all T cells (CD3^+^), cytotoxic T cells (CD8^+^), helper T cells (CD4^+^), natural killer cells (NKCs), circulating dendritic cells (cDC), macrophage/monocytes (Mac/Mon), and myeloid derived suppressor cells (MDSCs). **Figure 3A** displays our general gating strategies, and a table listing all gating markers is presented in **Supplementary Table S3** with representative gating for FcR in **Supplementary Figure S1**. Significant differences in relative populations of immune cells and in FcR were observed between the groups, as shown in the tSNE plots in **Figures 3B, C**.

When focusing on immune cell populations and comparing to TF, all three TB models showed significant decrease in B cells as a percentage of CD45+ cells, but only LLC TB mice displayed decreases in T cells and NKCs (**Figure 3D**). Notably, all T cell populations and NKCs were decreased in LLC TB mice compared to TF as well as compared to MC38 and CMT-167 TB mice. Cells derived from myeloid lineage illustrated different trends, however, where all three, cDCs, Mac/Mon, and MDSCs were upregulated in LLC TB mice compared to TF, but only cDCs and Mac/Mon were elevated in MC38 TB mice, and only Mac/Mon and MDSCs were elevated in CMT-167 TB mice compared to TF controls. Furthermore, all three cell populations were significantly higher in LLC compared to CMT-167, while only MDSCs were higher in LLC compared to MC38. cDCs and Mac/Mon were higher in MC38 compared to CMT-167, and only MDSCs were higher in CMT-167 compared to MC38. In summary, immune cell populations are significantly different in the four groups of mice, and the LLC TB mice with the most severe cachexia phenotype are notably different with lower T cell and higher MDSC populations compared to the other two models with mild (CMT-167) or no cachexia (MC38).

### 3.4 FcRn expression is significantly increased in immune cells in cachectic, LLC tumor-bearing mice

**Figure 4:**
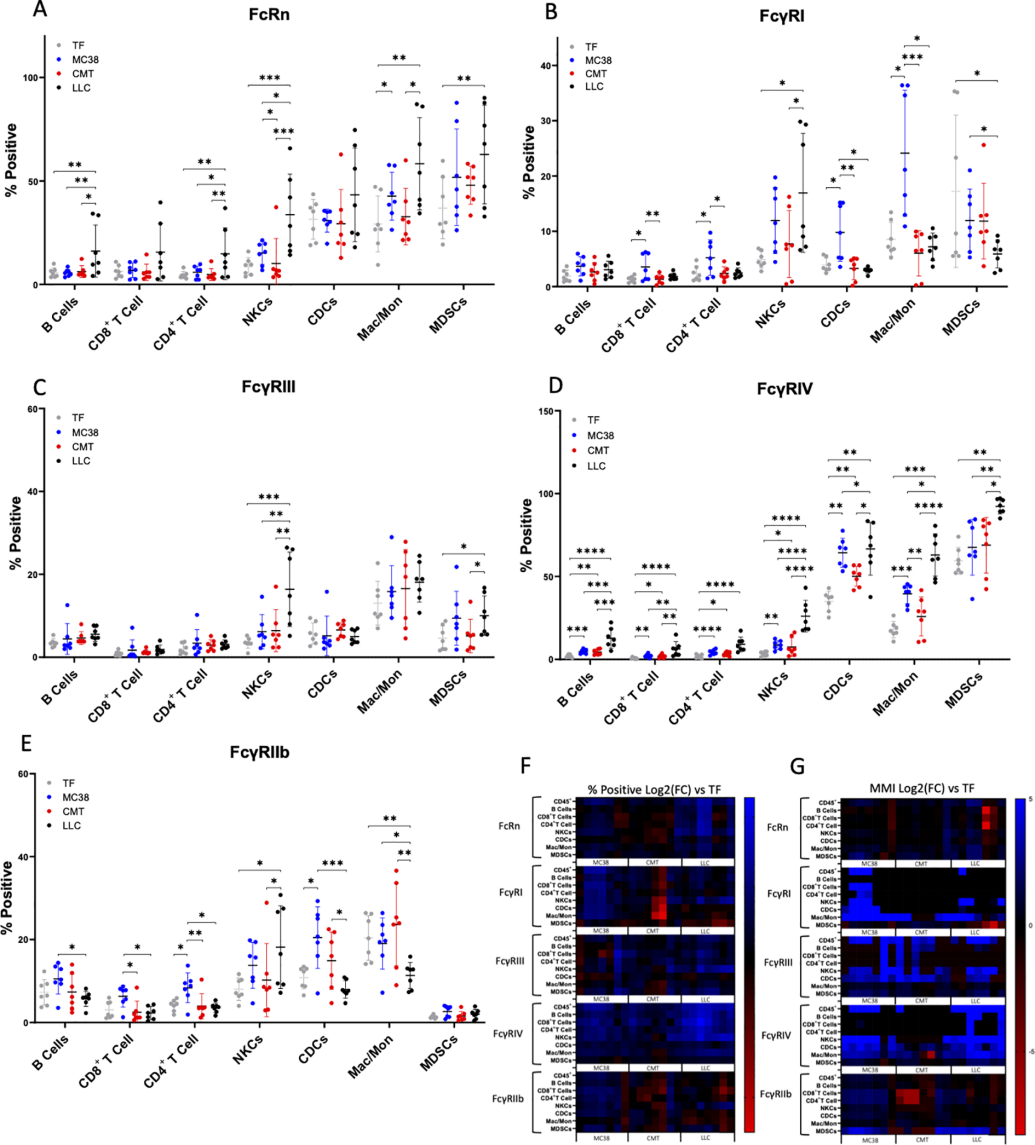
Fc Receptor expression changes as a function of tumor status and in correlation with skeletal muscle atrophy. Splenocytes from TF, MC38, CMT-167, and LLC (n=7 per group) analyzed by CyTOF for FcR expression on circulating immune cells expressed as % of subpopulation expressing the receptor (percent positve) for **(A)** FcRn **(B)** FcγRI **(C)** FcγRIII **(D)** FcγRIV and **(E)** FcγRIIb by ANOVA, Mean SD. Heat map illustrating **(F)** percent positive and **(G)** MMI Log_2_, (Fold Change) vs. tumor free, of immune cell subpopulation Fc receptor expression. *P<0.05; **P<0.01; ***P<0.001; ****P<0.0001 by ANOVA.

Due to FcRn’s role in IgG homeostasis, we hypothesized that elevated mAb CL in CMT-167 and LLC TB mice, and decreased FcRn gene (*Fcgrt*) expression in liver of LLC TB mice, as previously reported (16, 18, 35), would translate to decreased FcRn protein expression in immune cells of CMT-167 and LLC mice. However, our observations revealed the percentage of cells positive for FcRn staining (% pos), was significantly increased from TF in five (B Cells, CD4^+^ T Cells, NKCs, Mac/Mon, MDSCs) of the seven immune cell populations evaluated for LLC (**Figure 4A**). For MC38, the % pos for FcRn trended upward in some populations, but the difference was statistically significant in only the Mac/Mon population. For CMT-167, FcRn % pos was not different from TF in any cell population. Notably, when looking at median metal intensity (MMI), which is a measure of the signal intensity and therefore receptor density per cell, no statistically significant trends were observed for any of the three TB groups compared to TF (**Supplementary Figure S2A**).

### 3.5 FcγR expression differs between murine models of cancer and cancer cachexia

When looking at changes in expression of the activating FcγRs, we observed for FcγRI overall low % pos in TF mice, with MDSCs having the most abundant expression among all cell populations (**Figures 4B**, **S2B**). Compared to TF, percent positivity is higher for FcγRI in MC38 TB mice in all cell populations (% pos and/or MMI), except B cells and MDSCs. FcγRI expression in LLC TB compared to TF mice is higher in NKCs (% pos) and Mac/Mon (MMI) and lower in MDSCs (% pos and MMI) and was not different in any cell types for CMT-167 TB versus TF mice. For FcγRIII in TF mice, Mac/Mon (% pos) and MDSCs (MMI) had the highest expression, and significant differences from TF were only observed in LLC for NKCs and MDSCs (% pos), in MC38 for NKCs, cDCs, and MDSCs (MMI), and in CMT-167 for cDCs (MMI) (**Figures 4C**, **S2C**). FcγRIV in TF mice also displayed low expression on lymphoid cells, though it was relatively high in myeloid cells, especially in MDSCs and cDCs (**Figures 4D**, **S2D**). FcγRIV expression had a high degree of change in nearly all cell types among the three tumor bearing models for % pos and/or MMI, although in MDSCs, only LLC had higher expression compared to TF (both % pos and MMI). FcγRIV expression generally increased the most in LLC, followed by MC-38, then CMT-167.

For the inhibitory FcγRIIb, TF mice displayed the highest expression in Mac/Mon followed by cDCs and NKCs, with the lowest expression in MDSCs among all cell types (**Figures 4E**, **S2E**). Notably, expression on B-cells was similar to NKCs, and even T cell expression was higher than on MDSCs. FcγRIIb expression trended higher in MC38 TB mice for all cell types, except Mac/Mon and MDSCs but was only significantly higher than TF mice in CD4+ T Cells (% pos) and CDCs (% pos and MMI). Opposite the trends in MC-38, FcγRIIb expression in LLC TB mice trended lower than in TF mice in most cell types, was significantly lower in Mac/Mon (% pos and MMI) and significantly higher in NKCs (% pos) and MDSCs (MMI). FcγRIIb was significantly increased (MMI) on Mac/Mon in CMT-167 TB compared to TF mice, but no other significant differences were observed in this tumor model for FcγRIIb. A heat map depicting fold change in FcR expression across models is illustrated in **Figures 4F, G**.

### 3.6 FcR expression changes correlate with skeletal muscle mass and baseline ICI CL in patients receiving ICI therapy

**Figure 5:**
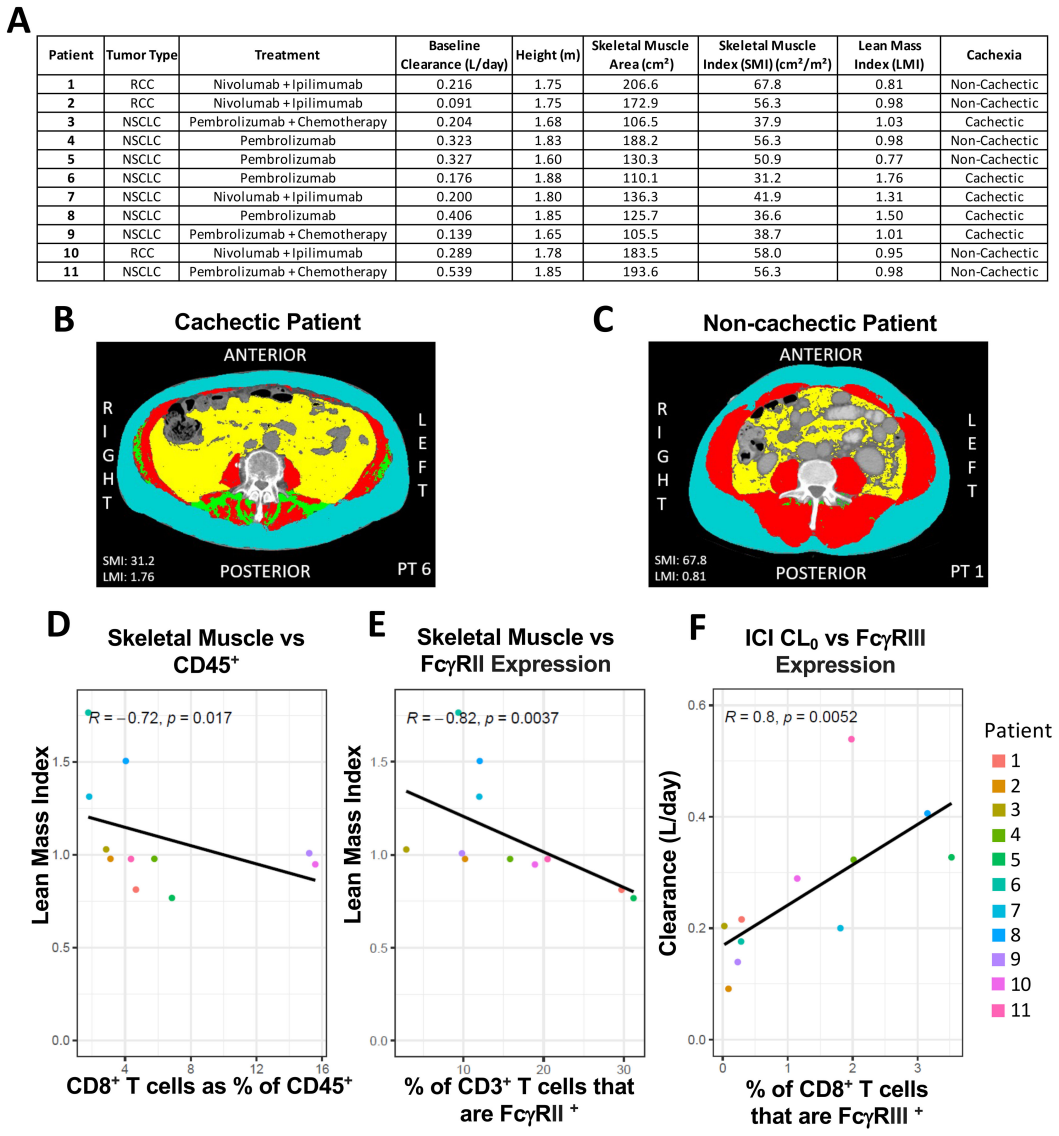
Patient skeletal muscle mass correlates with immune cell and FeR expression. Clinical patient L3 cross sectional CT scan analyzed for skeletal muscle surface area and normalized to height, and sex dependent thresholds. Patient whole blood prior to ICI treatment was collected and stained for CyTOF analysis of immune cells and FcR expression. Patient baseline ICI mAb clearance (CL_0_) was estimated and analyzed against CyTOF. **(A)** Summary of characteristics from patients included in CyTOF analysis and calculation of Lean Mass Index. Representative L3 cross sectional CT scan analyzed for skeletal muscle surface area for a patient with **(B)** low Lean Mass Index and **(C)** high Lean Mass Index. Representative linear regression plots in clinical population depicting only raw (non adjusted) p value **(D)** LMI vs. circulating levels of CD8^+^ T cells expressed as % of total CD45^+^**(E)** LMI vs. % of CD3^+^ T cells that are FcγRII^+^**(D)** Patient ICI CL_0_ vs. % of CD8^+^ T cells that are FcγRII^+^ positive.

To determine if the observations in our murine models are representative of immune cell and FcR expression patterns associated with body composition and disease state in cancer patients, we completed a similar analysis in a small, clinical population of 11 subjects. Patients with either RCC or NSCLC receiving pembrolizumab (±chemotherapy) or nivolumab (+ipilimumab) were enrolled in a non-interventional study. Patient demographic and treatment information is provided in **Figure 5A**. Patient whole blood was collected at baseline on cycle 1 day 1 of ICI therapy, stained with metal-labeled antibodies, and separated PBMCs analyzed by CyTOF. Our primary analysis focused on major populations listed in **Supplementary Table S4** with representative gating shown in **Supplementary Figures S3**, **S4**. Patient baseline L3 vertebrae CT images were analyzed to assess skeletal muscle surface area according to pre-published methodology (**Figures 5B, C**) (38). In clinical populations, cachectic phenotype presents on a spectrum of severity. In order to better evaluate the relationship between extent and severity of cancer associated skeletal muscle loss we aimed to evaluate cachectic phenotype in patients on a continuous scale of Lean Mass Index (LMI). LMI normalizes patient skeletal muscle surface area to published and validated cutoffs (40, 41). LMI was derived as described in the methods. A LMI >1 indicates increasing severity of skeletal muscle depletion (**Figure 5B**), while LMI <1 indicates increasing skeletal muscle mass above the established sex-dependent thresholds (**Figure 5C**). Immune cells, PD-1, and FcR expression were analyzed against LMI and CL_0_ in order to identify positive (green) and negative (yellow) correlations between body composition, ICI CL, and receptor expression (**Figures 5D–F**; **Table 1**). 348 individual Spearman’s rank correlation analyses were conducted in this population, due to the large size of analyses only notable trends (raw p value <0.05) were reported in **Table 1**. Multiple hypothesis testing was adjusted using Benjamini-Hochberg (BH) correction; however, due to the large number of comparisons, no correlation retained a BH Adjusted p Value of < 0.05. Nonetheless, these exploratory findings remain translational relevant and complement similar analysis reported in murine models of cachexia, although confirmation in a larger dataset will be required. Nivolumab and pembrolizumab clearance rates were analyzed together, as there was no significant difference in CL_0_ between treatment groups (**Supplementary Figure S5**). While this clinical dataset represents a sparse and heterogenous patient population, it offers a glimpse into changes of Fc receptor and immune cell expression in correlation with skeletal muscle mass and ICI CL in cancer patients. There were notable correlations observed between patient LMI and changes in immune cells as a percent of CD45^+^ cells (**Table 1**). The most notable being the negative relationship between lean mass and CD8^+^ T cells, suggesting that decreased lean mass correlates with reduced circulating cytotoxic T cells. Additionally, negative correlations were observed between LMI and FcγRII expression on T cells (CD3^+^, CD8^+^, Th2) as well as plasmacytoid DCs (pDC). LMI and FcRn expression had a positive relationship on many cell types (**Table 1**; **Supplementary File S1**), suggesting that decreased lean mass correlates with increases in FcRn expression in circulating leukocytes. There were also observed correlations between LMI and FcγRI, FcγRIII, and PD-1 expression on various immune cells. Patient CL_0_ for respective therapy was then analyzed against receptor and immune cell expression in baseline patient PBMCs (**Table 1**). There was not a strong relationship observed between body composition and CL_0_ in this limited dataset. Interestingly there was a positive relationship between CL_0_ and FcγRI, FcγRII, and FcγRIII expression on immune cells. There were no notable (raw p value <0.05) relationships between ICI CL_0_ and FcRn expression in immune cells (**Table 1**). The full correlation analysis dataset is available in the supplement (**Supplementary File 1**).

**Table 1:** 

Variable	Receptor	Covariate	R Value	Raw p Value	BH Adjusted p Value
Lean Mass Index (Cachexia)	**% CD45+**	CD8^+^ (% pos)	-0.72	0.017	0.14
		NK cells (% pos)	-0.68	0.025	0.14
		Non classical monocyte (% pos)	-0.67	0.028	0.14
	**RI**	Non classical monocyte (% pos)	-0.64	0.040	0.35
		Classical monocyte (% pos)	-0.62	0.048	0.35
	**RII**	CD3^+^ (% pos)	-0.82	0.004	0.07
		CD8^+^ (% pos)	-0.81	0.004	0.07
		Th2 (% pos)	-0.76	0.009	0.10
		Plasmacytoid DC (% pos)	-0.69	0.018	0.13
		CD8^+^ (MMI)	-0.70	0.021	0.13
	**RIII**	CD45^+^ (MMI)	-0.65	0.031	0.99
	**FcRN**	Monocyte derived DC (MMI)	0.70	0.031	0.19
		Monocytic MDSC (% pos)	0.72	0.024	0.19
		Monocytic MDSC (% pos)	0.72	0.024	0.19
		Non classical monocyte (MMI)	0.65	0.049	0.19
		NK cells (MMI)	0.64	0.047	0.19
		Monocyte derived DC (% pos)	0.76	0.016	0.19
	**PD-1**	Granulocytic MDSC (% pos)	0.71	0.019	0.30
		Monocyte derived DC (MMI)	-0.85	0.002	0.07
Baseline ICI Clearance	**RI**	CD45^+^ (MMI)	0.77	0.005	0.16
		Monocyte derived DC (MMI)	0.72	0.017	0.16
		Non classical monocyte (MMI)	0.70	0.021	0.16
		Th17 (% pos)	0.68	0.022	0.16
		Classical monocyte (% pos)	0.67	0.028	0.17
	**RII**	CD8^+^ (MMI)	0.77	0.008	0.26
		CD3^+^ (% pos)	0.68	0.025	0.33
		CD8+ (% pos)	0.66	0.031	0.33
	**RIII**	CD8^+^ (% pos)	0.80	0.005	0.13
		Th1 (% pos)	0.73	0.011	0.13
		CD4^+^ (% pos)	0.74	0.013	0.13
		CD3^+^ (% pos)	0.71	0.019	0.13
		Th2 (MMI)	0.70	0.021	0.13
		CD8^+^ (MMI)	0.66	0.031	0.15
		T Regulatory (% pos)	0.64	0.032	0.15
	**PD-1**	Monocyte derived DC (MMI)	0.65	0.037	0.57

### 3.7 Cachexia induced by Activin A/IL-6 does not alter ICI Clearance or FcR expression

**Figure 6:**
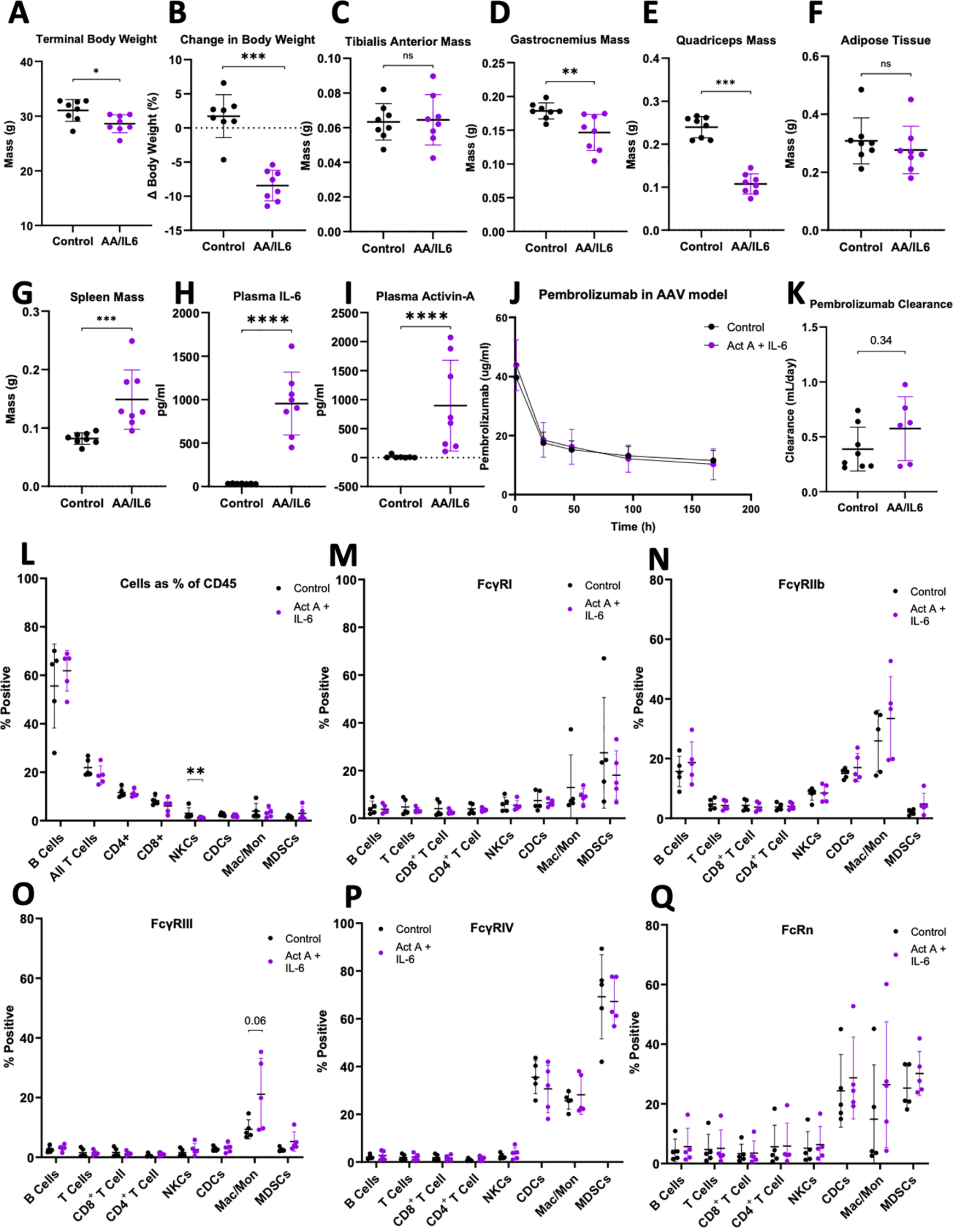
Adeno-Associated Virus model of induced skeletal muscle atrophy does not alter ICI PK or FcR expression. C57BL/6 mice aged 20 weeks old were randomly assigned into groups of control vector (Control; n=8) or active AAV (AA/IL6 or ActA+IL-6; n=8) with viral vector encoding for the production of Activin-A (1x10^12^ viral vector genomes) and IL-6 (2.6x10^10^ viral vector genomes). On day 0, mice were injected with half of the total viral dose into the right quadricep, with the other half in the left quadricep. Mouse body weights were measured every 7 days. 12 weeks post inoculation mice were euthanized and assessed for cachexia phenotype **(A)** Terminal bodyweight **(B)** Change in body weight over the study time course, terminal bodyweight expressed as a percentage of initial (day-1) bodyweight **(C)** Tibialis Anterior mass **(D)** Gastrocnemius mass **(E)** Quadriceps mass **(F)** Epididymal Adipose Tissue mass **(G)** Spleen mass. Measurement of terminal plasma levels of **(H)** IL-6 and **(I)** Activin-A by Mann-Whitney Test. **(J)** At 8 weeks post inoculation, a single i.v. dose of pembrolizobmab was administered and plasma pembrolizumab concentration vs time between control and ActA+IL-6 groups was measured. **(K)** NONMEM derived individual pembrolizumab clearance estimates by Wilcoxin-Signed Rank Test. CyTOF analysis of control and ActA+IL-6 mouse splenocytes, **(L)** Circulating immune cell populations as % of CD45^+^. % pos expression on circulating immune cells for **(M)** FcRn **(N)** FcγRI **(O)** FcγRIII **(P)** FeγRIV **(Q)** FcγRIIb by Mann-Whitney Test. Mean ± SD; *P<0.05; **P<0.01; ***P<0.001; ****P<0.0001.

A previously published Adeno-Associated Virus (AAV) model of induced skeletal muscle atrophy and inflammatory skeletal muscle wasting was utilized to investigate the effects of body composition changes on ICI PK and FcR expression in the absence of a tumor (34). Mice were injected with either AAV containing viral vector encoding for IL-6 and Activin-A (n= 8, ActA+IL-6) or an AAV with empty vector control (n=8, control) (17). ActA+IL-6 mice displayed a phenotype consistent with the description of skeletal muscle atrophy with decreased body weight (**Figures 6A, B**) and muscle mass (**Figures 6C–E**). There was no observed difference in adipose tissue (**Figure 6F**), though spleen mass was larger vs. control (**Figure 6G**). As expected, ActA+IL-6 mice displayed significantly elevated levels of plasma Activin-A and IL-6 compared to control virus injected mice (**Figures 6H, I**). Mice enrolled in the study were administered a single intravenous dose of pembrolizumab, and mice displaying apparent absorption of injected antibody were excluded from pharmacokinetic analysis. No difference in pembrolizumab concentration vs time was observed (**Figure 6J**), and estimated clearance of pembrolizumab was not different in ActA+IL6 versus control mice (**Figure 6K**; p=0.44).

In order to assess the effect of ActA+IL-6 induced skeletal muscle atrophy upon immune cell populations and their respective Fc Receptor expression profiles, randomly selected splenocytes from control mice (n=5) and ActA+IL-6 mice (n=5) were analyzed by CyTOF. Despite significant changes in body composition, skeletal muscle, and spleen, there were negligible changes in expression of immune cells as a percent of total CD45^+^ (**Figure 6L**). Further, when analyzing FcR expression on circulating immune cells there were no statistically significant changes in either % pos (**Figures 6M–Q**) or MMI (**Supplementary Figure S6**) between control and AAV mice.

## 4 Discussion

With the goal of understanding the relationships between FcR expression, mAb CL, and body composition/cachexia phenotype, this study is the first report investigating expression changes of FcRs and FcR-expressing immune cell populations in tumor-bearing mice and in patients with cancer who are receiving ICIs. The analyses of splenocytes from preclinical models illustrate distinct differences in FcR expression between tumor models that induce minimal, mild and severe forms of cancer cachexia and that lead to differing levels of catabolic IgG mAb clearance. Similarly, in patients with NSCLC and RCC, we observe apparent correlations between FcR expression in subpopulations of circulating immune cells, baseline systemic ICI clearance, and lean body mass.

As peripheral immune cells, leukocytes are important mediators of immune surveillance and subsequent ICI efficacy, and immune cell changes can be predictive markers of OS in ICI therapy (47, 48). When looking at differences in immune cell populations in TB mice (MC38, CMT-167, LLC) compared to TF mice, as a % of total CD45^+^, the trends were generally similar across the tumor types, with decreases in the proportion of B cells and increases in the proportion of CDCs and Mac/Mon. The LLC murine model of severe cancer cachexia demonstrated some of the most distinct differences in immune cell populations compared to TF mice and even compared to non-cachectic MC38 and mildly cachectic CMT-167 TB mice, such as decreases in CD8^+^ and CD4^+^ T cells and NKCs. Notably, while the proportion of MDSCs was not different in MC38 TB vs TF mice, MDSCs increased significantly in the mildly cachectic CMT-167 model and even more so in the severely cachectic LLC model. These changes in the MDSC population across tumor models highly corresponds with cachexia phenotype and to some extent, the elevated IgG mAb CL observed in both CMT-167 and LLC TB vs TF mice, but not in MC38 TB mice. The sharp increase in MDSCs could conceivably contribute toward immune suppression and inflammation associated with cachexia syndrome (49). Patient PBMCs showed similar trends as decreases in patient lean mass correlated with decreased levels of CD8^+^ T cells and NKCs. Reduced levels of CD8^+^ cytotoxic T cells at time of treatment initiation could potentially affect therapeutic outcomes as cytotoxic T cells are imperative for checkpoint inhibition (47, 48).

With respect to FcRn, we hypothesized observed elevated mAb CL in CMT-167 and LLC TB mice and decreased *Fcgrt* expression in liver of LLC TB mice (16, 18, 35) would reveal decreased FcRn protein expression in immune cells of CMT-167 and LLC mice. However, our results demonstrate no evidence of reduced FcRn expression in any of the TB models compared to TF, and in fact suggest the percent of cells positive for FcRn expression is increased in multiple cell types (B Cells, CD4^+^ T Cells, NKCs, Mac/Mon, MDSCs) in splenocytes of LLC TB mice and in Mac/Mon of MC38 TB mice (**Figure 4A**). However, we did not observe any differences in MMI for any cell types across the three tumor models compared to TF mice, suggesting that while the proportion of cells detected as positive for FcRn expression may be higher in several immune cell populations in LLC TB mice, there was no evidence that expression of FcRn significantly increases per cell within each population (**Supplementary Figure S2A**). Given FcRn’s role in IgG homeostasis, these results are counter to our hypotheses, and they demonstrate no clear trends that would help to explain elevated IgG mAb CL in both LLC and CMT-167, but not in the MC-38 TB models compared to TF mice. Similarly, when evaluating FcRn expression in patient CD45^+^ PBMCs, no correlations were observed with ICI mAb CL_0_ (**Table 1**). We do note however that FcRn expression in some PBMC immune populations does correlate with lean mass index, which reflects cachexia phenotype (**Table 1**). While the observed increases in FcRn expression in circulating immune cells in both mice and humans with cancer cachexia are counter to our hypotheses, these results are consistent with our prior findings in murine models pancreatic cancer and in circulating immune cells of patients with pancreatic cancer (37). Relatively little is known concerning regulators of FcRn expression (22), however our data would suggest, at least in mice, that neither elevated circulating IL-6 or activin A have direct effects on immune cell FcRn expression. The notable alterations in FcRn levels coinciding with skeletal muscle atrophy warrants further investigation into regulation of FcRn expression and function, and impact on IgG catabolism and efficacy of antibody therapeutics. Furthermore, while FcRn expression in circulating immune cells does not appear to be an explanation for the observed elevation in catabolic CL, FcRn function has not been assessed in the context of cancer cachexia. Furthermore, expression of FcRn protein may differ in endothelial cells, which represent another key compartment for IgG uptake and homeostasis. Therefore, functional studies of FcRn and expanded evaluation of FcRn in endothelial cells of various tissues will be warranted.

With respect to activating FcγRs, FcγRIV displayed the largest difference in expression in murine splenocytes, with LLC mouse leukocytes displaying the most drastic increase, CMT-167 the least dramatic increase, and MC-38 falling in between for FcγRIV levels compared to TF mice (**Figure 4D**, **Supplementary Figure S2D**). Increases in activating FcγRIV expression has the potential to increase effector functions depending on cell type, but FcγRIV primarily facilitates phagocytosis/cytotoxicity, which is important for the clearing of malignant cells (50, 51). Further, anti-PD-1 efficacy is potentiated in mice lacking functional activating FcγRs (I, III, and IV) (25). We also detected high expression of FcγRIV on LLC MDSCs (>90%), but there exist no prior reports of FcγRIV expression or its function in this cell type. The human functional ortholog of mFcγRIV, CD16 or hFcγRIII, is important for the activity of anti-tumor targeted IgG1 antibodies (52, 53). In patient PBMCs, a slightly negative trend exists between lean mass index (cachexia phenotype) and the hFcγRIII MMI of CD45^+^ cells (**Table 1**). Similarly, comparing patient CL_0_ rates to hFcγRIII expression, there is a positive correlation in nearly all cell types, including notable correlations in seven T cell subpopulations. Given the receptor’s role in phagocytosis/cytotoxicity it suggests that activating FcγRs on immune cells and changes in FcγR expressing immune cells are associated with alterations in Fc effector functions that could impact ICI behavior in patients.

Similar to FcγRIV, murine FcγRIII also displayed the most significant increases in LLC TB compared to TF mice, but only for % pos in NKCs and both % pos and MMI in MDSCs. FcγRIII MMI was also higher in MC-38 TB mice on NKCs, CDCs, and MDSCs. Immune cells from splenocytes of MC-38 TB mice also displayed the most dramatic difference for the remaining activating receptor, FcγRI, which was significantly higher on all cell types (% pos and/or MMI) except B cells and MDSCs. Interestingly, in LLC TB mice, FcγRI was increased on NKCs and Mac/Mon but decreased on MDSCs. In patient PBMCs, we observed correlations between FcγRI expression, lean mass index, and CL_0_, predominantly in myeloid cell populations with Th17 cells as the one exception.

Alterations in the balance of activating and inhibitory FcγRs can perturb downstream signaling and the cell’s threshold of activation for Fc effector functions (28, 29). Deviations from physiological expression of FcγRs have been shown to correlate with disease severity in some autoimmune disorders (29, 54, 55), yet thus far the importance of relative activating to inhibitory signaling for ICI therapy has only been studied in the context of comparing mAb IgG isotypes, which dictates a preferential binding for the various FcγR isoforms (23). This predisposition in IgG backbone, as it relates to preferential binding of either activating or inhibitory receptors, drives anti-tumor outcomes in some anti-PD-1 and anti-PD-L1 treatments (25, 30). With respect to FcγRIIb, the sole inhibitory receptor, we observed MC-38 TB mice to have the most dramatic increase relative to TF in almost all cell types. This receptor was also the most variable among the different murine tumor models whereby trends for decreasing expression were observed in B cells and T cells for LLC and CMT-167, significantly decreased vs. increased expression in Mac/Mon for LLC and CMT-167, respectively, and significantly increased expression in NKCs and MDSC for LLC only. Collectively, these results indicate alterations in FcγR expression as well as alterations in the balance between activating and inhibitory receptors and presumably signaling across the different tumor models. The altered expression of FcγRs and activating/inhibitory signaling in the context of disease state or across tumor types, as observed in our data, could impact Fc effector functions differently, including ICI internalization and catabolism, and ultimately efficacy (25, 28, 29, 51, 54, 55). While an in-depth understanding of FcR expression changes is important, future studies will be needed to determine how these changes in FcR expression, both individual cell-type contributions and their combined effects, impact FcR functions *in vivo*, and ultimately immune outcomes.

Further, there were many notable, positive relationships observed between patient ICI CL_0_ and FcγR expression on T cell subsets. It is unclear how T cell FcγR expression could affect antibody catabolism, however its strong correlation with ICI CL_0_ in many T cell subtypes indicate its potential utility as a biomarker of clearance and therefore, outcomes. It is important to acknowledge that these relationships express only observed correlations in this small patient population as opposed to evidence of a causative relationship. These results identify a gap in knowledge and a need for studies exploring how changes and/or expression signatures in patient circulating immune cells may either directly affect ICI PK and efficacy or serve as potentially useful prognostic or predictive markers of outcomes.

It is notable that in nearly all cases, when FcR expression differences were observed relative to TF mice, the difference was an increased expression. The rare exceptions were for LLC TB mice, which displayed lower expression of FcγRI on MDSCs and FcγRIIb on Mac/Mon. It is also notable that among the three tumor models, the CMT-167 model with mild cachexia and elevated mAb CL had the least dramatic change of Fc receptors in all cell types compared to TF mice. We observed no differences for FcRn or FcγRI, only in cDCs (MMI) for FcγRIII, and while it differed in nearly all cell types for FcγRIV, the observed differences vs. TF mice were the least among the three tumor models. Similarly, for the inhibitory receptor, FcγRIIb, expression trended both up and down across cell types, but the only significant difference was an increased MMI on Mac/Mon.

We highlight here that since mAb catabolic CL occurs in all tissues, and since cachexia wasting syndrome occurs distal to the tumor site and varies among tumor type and tumor location, we focused on the impact of tumor and/or cachexia on circulating host immune cell populations and their characteristics, without evaluating tumor-intrinsic properties. However, we acknowledge tumor properties, including localized effects in tumor infiltrating leukocytes (TILs) and their FcR phenotype will be important for future study.

A major obstacle in cancer cachexia research is the challenge of decoupling the contributions of the tumor mass itself, versus other factors, with respect to the cachexia phenotype. The AAV model allows for the assessment of cytokine-driven skeletal muscle atrophy on ICI CL and immune cell FcR expression profiles in the absence of tumor. Mice receiving viral vector encoding for IL-6 and Activin A demonstrated a phenotype consistent with cachexia, displaying reduced body weight and reductions in skeletal muscle mass. It was hypothesized that these changes in body composition would coincide with increased pembrolizumab clearance, though this was not observed. It was further hypothesized that skeletal muscle atrophy in the AAV mice would mimic tumor-mediated inflammation, promote immune cell expansion, and alter FcR expression on various cell types. However, there were no observable differences in relative sizes of immune cell populations nor FcR expression between groups, despite significant splenomegaly and altered body composition. These results suggest that increased clearance in murine models of cancer cachexia is not driven by body composition changes alone but instead is driven by tumor-host interactions that manifest as the cancer cachexia phenotype. This study also implies that the specific FcR expression profiles observed in tumor-bearing models may also be dependent on the tumor itself, since the AAV model displayed no modulation in FcR expression, and FcR expression was different across all the tumor types evaluated. Given that these results were both dependent on tumor type and presence, suggest the need for further understanding of the tumor intrinsic properties dictating alterations in body composition, ICI CL, and FcR expression. While the AAV model was unable to replicate changes seen in murine models of cancer cachexia it will be important in the future to study other models of inflammation induced skeletal muscle wasting and other models of cachexia, such as kidney injury or sepsis, and their impact on ICI CL and FcR expression. These studies will provide valuable understanding of the factors and pathways driving cachexia associated changes in antibody catabolism and FcR expression in the absence of tumor burden.

When interpreting these results, there are some limitations to be considered that may affect the conclusions drawn from these studies. The clinical population included in the exploratory analysis of FcRs and immune cell expression correlations with body mass and ICI CL is a sparse, limited, and heterogenous sample population. Future studies exploring the relationship between cachexia, ICI CL, and FcR expression should build upon this work in a larger, more homogenous population with greater statistical power. Additionally, it is important to emphasize that the work contained within this report looked at the observed relationships between cachexia/body composition changes, ICI CL, and FcR expression. While these observed correlational changes are a foundational first step, future studies will be needed to validate and better understand the functional relationships between cachexia, changes in FcR expression, and ICI CL as it relates to treatment outcomes.

## 5 Conclusion

Patients with cancer cachexia generally display increased ICI CL, and this increased clearance serves as a biomarker of poor outcomes of ICI therapy irrespective of dose and drug exposure (3, 9, 11–13, 15). It was observed in pre-clinical and clinical populations that FcRn and activating FcγR expression show no change or increase, while inhibitory FcγRIIb expression in spleen decreases in correlation with losses in skeletal muscle and lean mass in cancer. There are also observed decreases in CD8^+^ T cells associated with skeletal muscle atrophy in a clinical population. When comparing patient ICI CL_0_ with FcR expression there were positive relationships between clearance and FcγR expression on certain T cell populations, though surprisingly CL was not correlated with FcRn expression. These results were not replicated in a tumor-free model of cachexia, as there was no impact on ICI CL, circulating immune cell populations, nor FcR expression in the IL-6/Activin A induced cachexia model relative to AAV vector control. This suggests that skeletal muscle atrophy alone is not sufficient to drive increased ICI CL and FcR expression changes, and instead implies these alterations are intrinsic properties of tumor-host interactions. Changes in FcγR expression as a function of disease state may impact antibody therapy across a range of disease modalities. Further study of disease-mediated FcR expressional changes, mechanisms driving these changes, and their impact on ICI PK and efficacy is warranted and may provide prognostic or predictive insight for ICIs and other antibody therapeutics.

## Data Availability

The raw data supporting the conclusions of this article will be made available by the authors, without undue reservation.
