# Supplementary figures and images for: Tumor intrinsic properties dictate Fc receptor expression and cancer cachexia associated increase in checkpoint inhibitor clearance
Source: Front Immunol. 2025 Dec 17;16:1669979. doi: 10.3389/fimmu.2025.1669979 (PMC12753407; doi:10.3389/fimmu.2025.1669979)

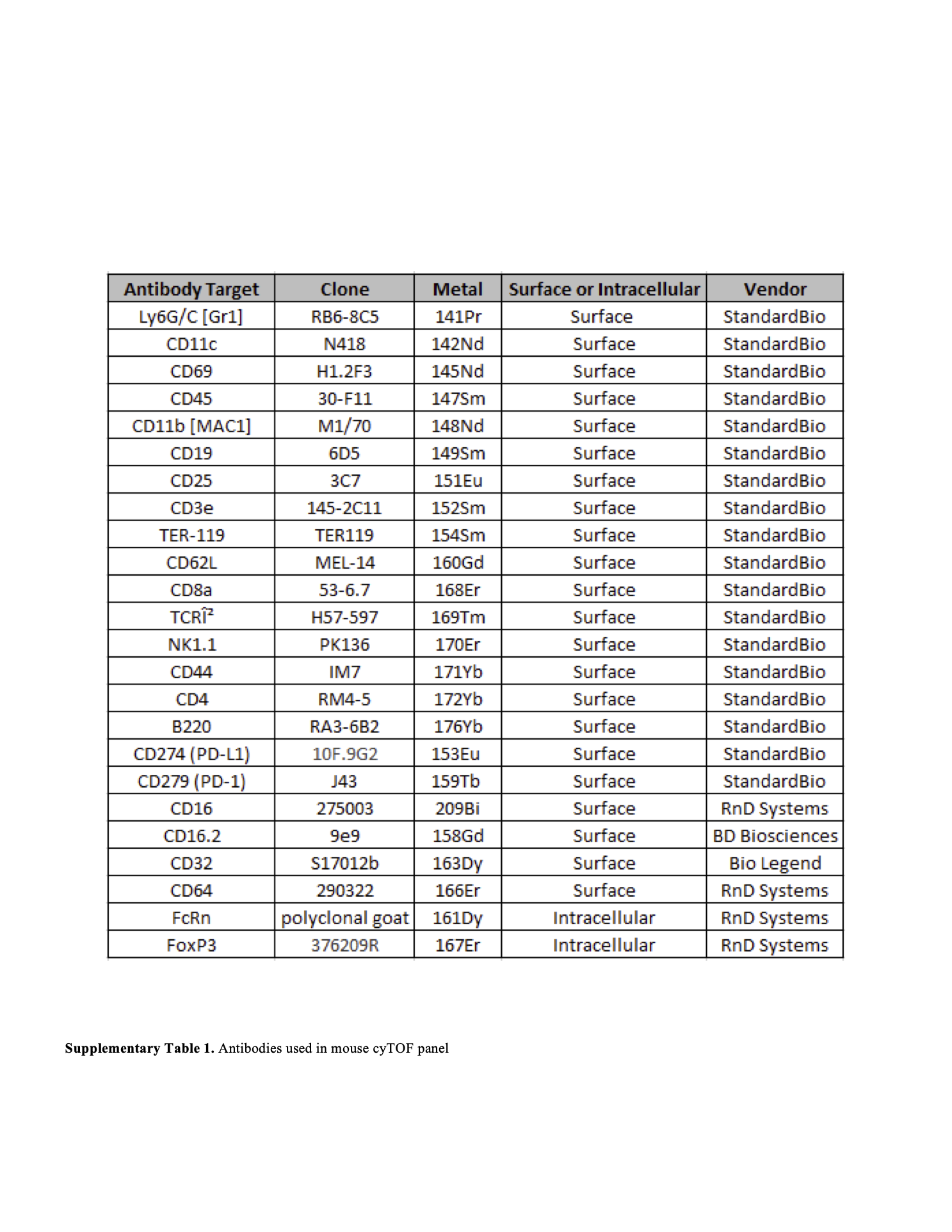

Supplement: Supplementary file 2 [file Image1.tiff]

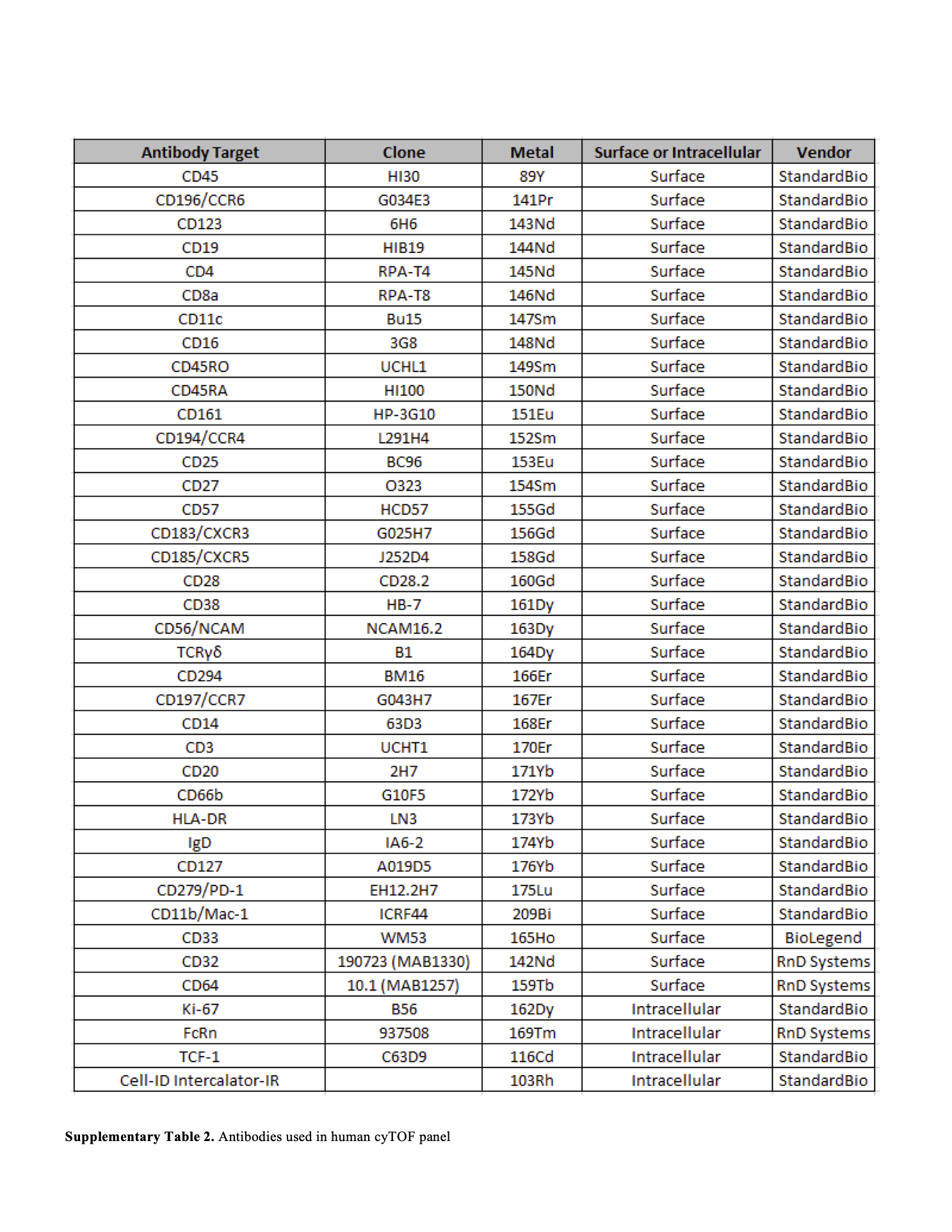

Supplement: Supplementary file 3 [file Image2.tiff]

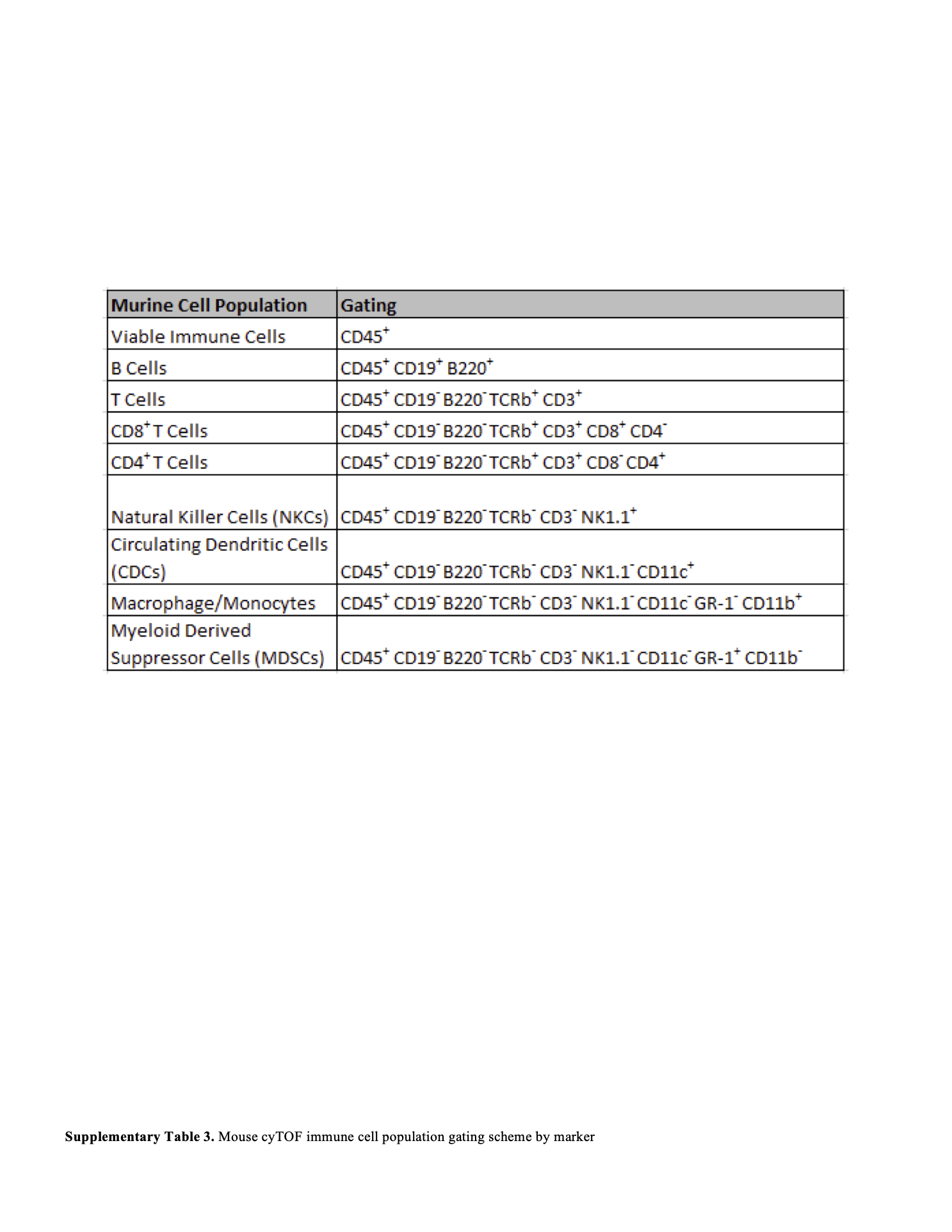

Supplement: Supplementary file 4 [file Image3.tiff]

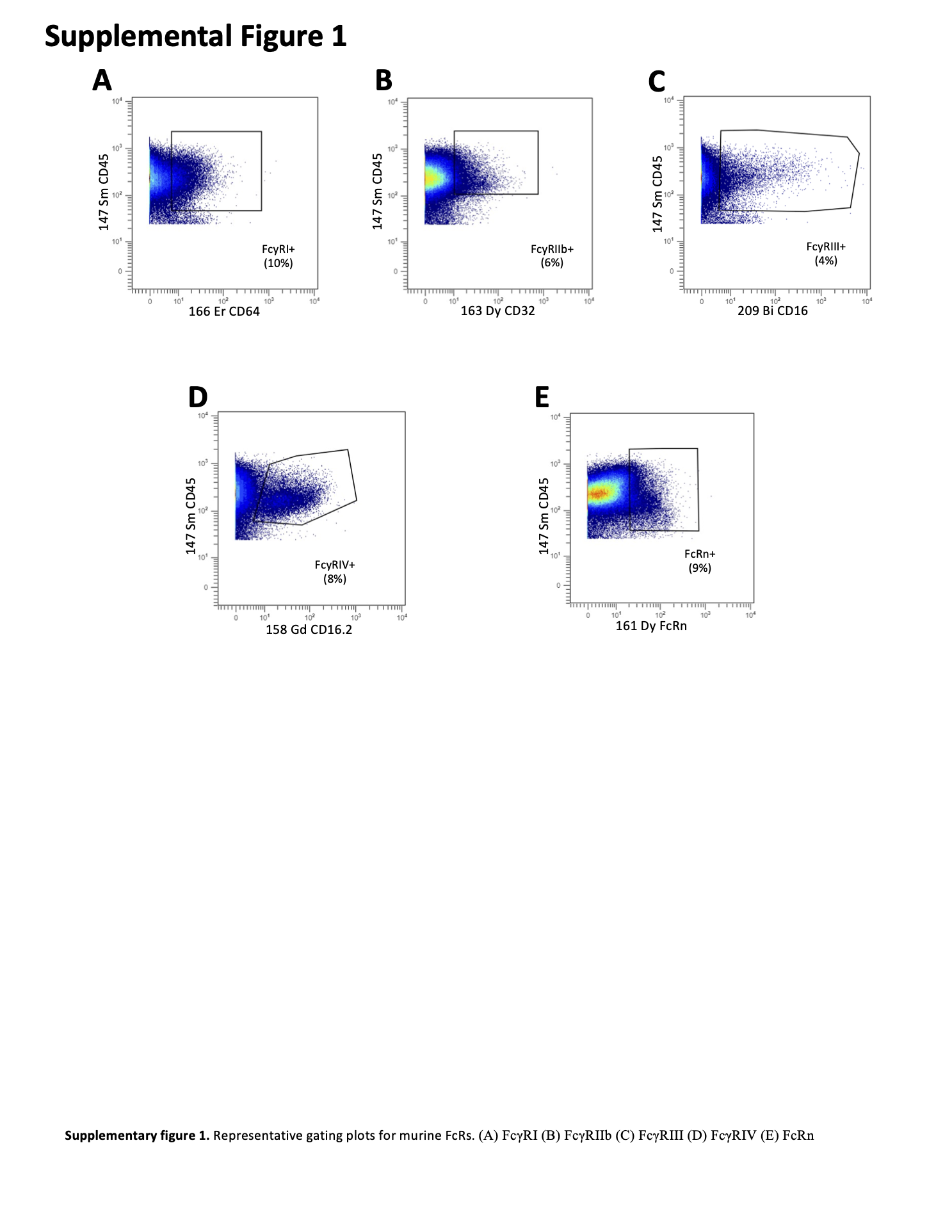

Supplement: Supplementary file 5 [file Image4.tiff]

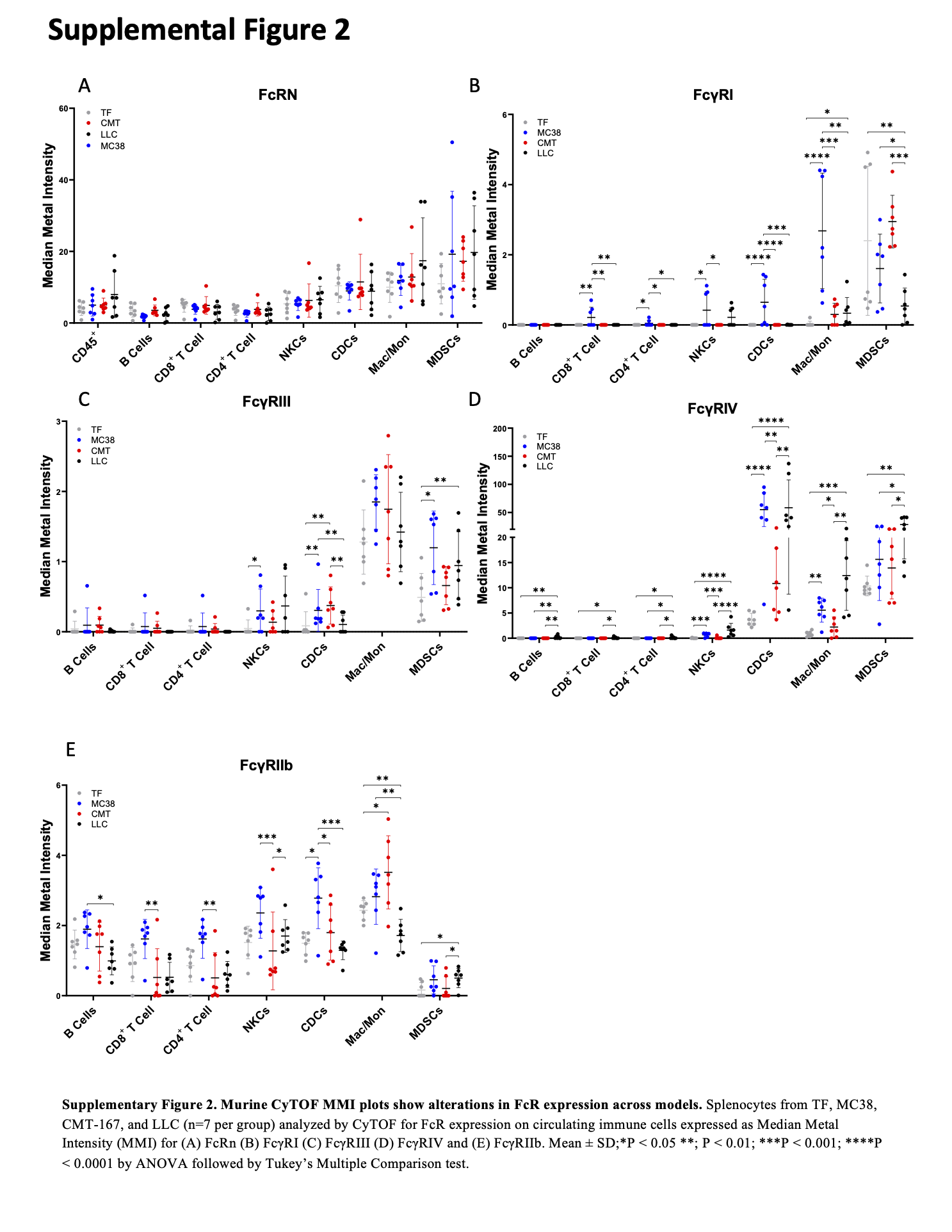

Supplement: Supplementary file 6 [file Image5.tiff]

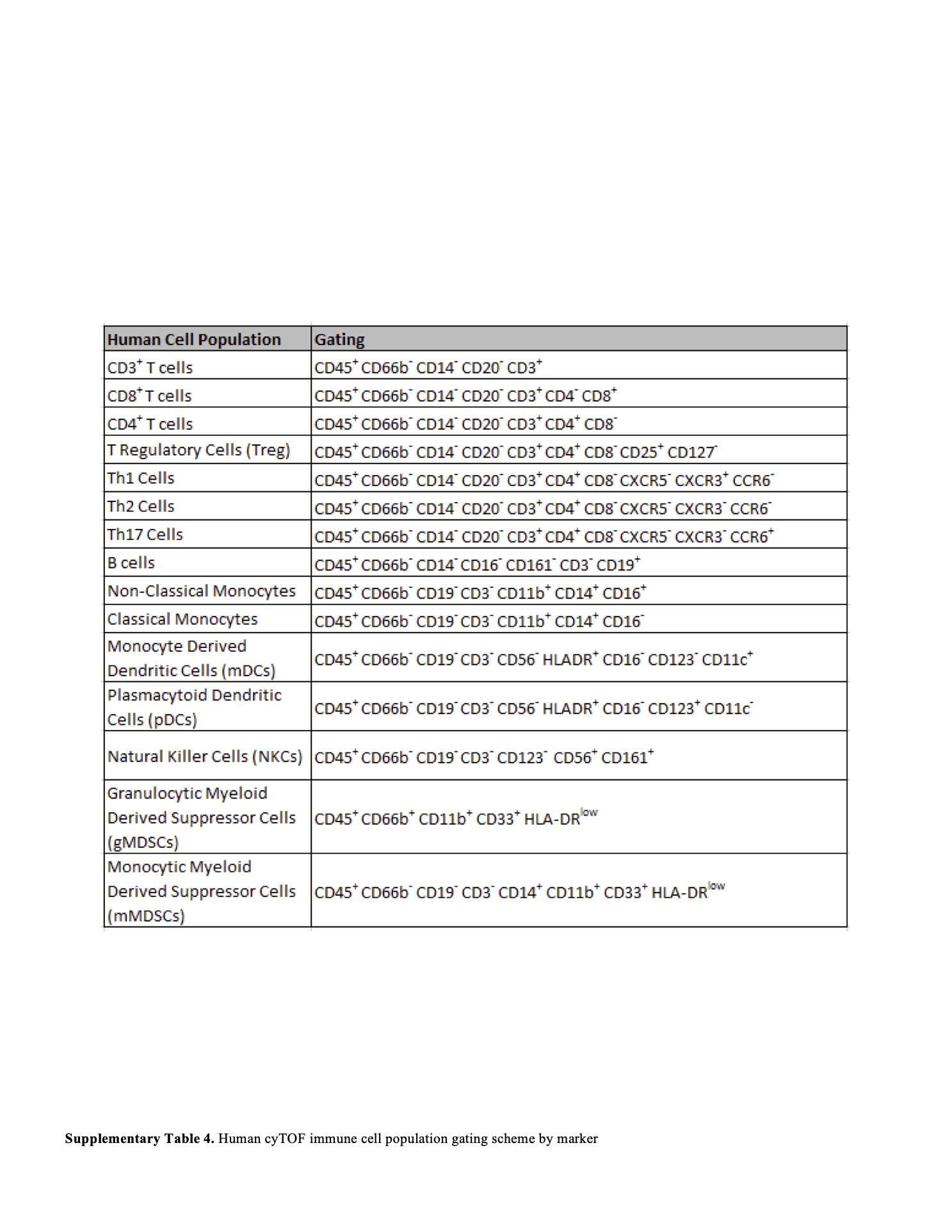

Supplement: Supplementary file 7 [file Image6.tiff]

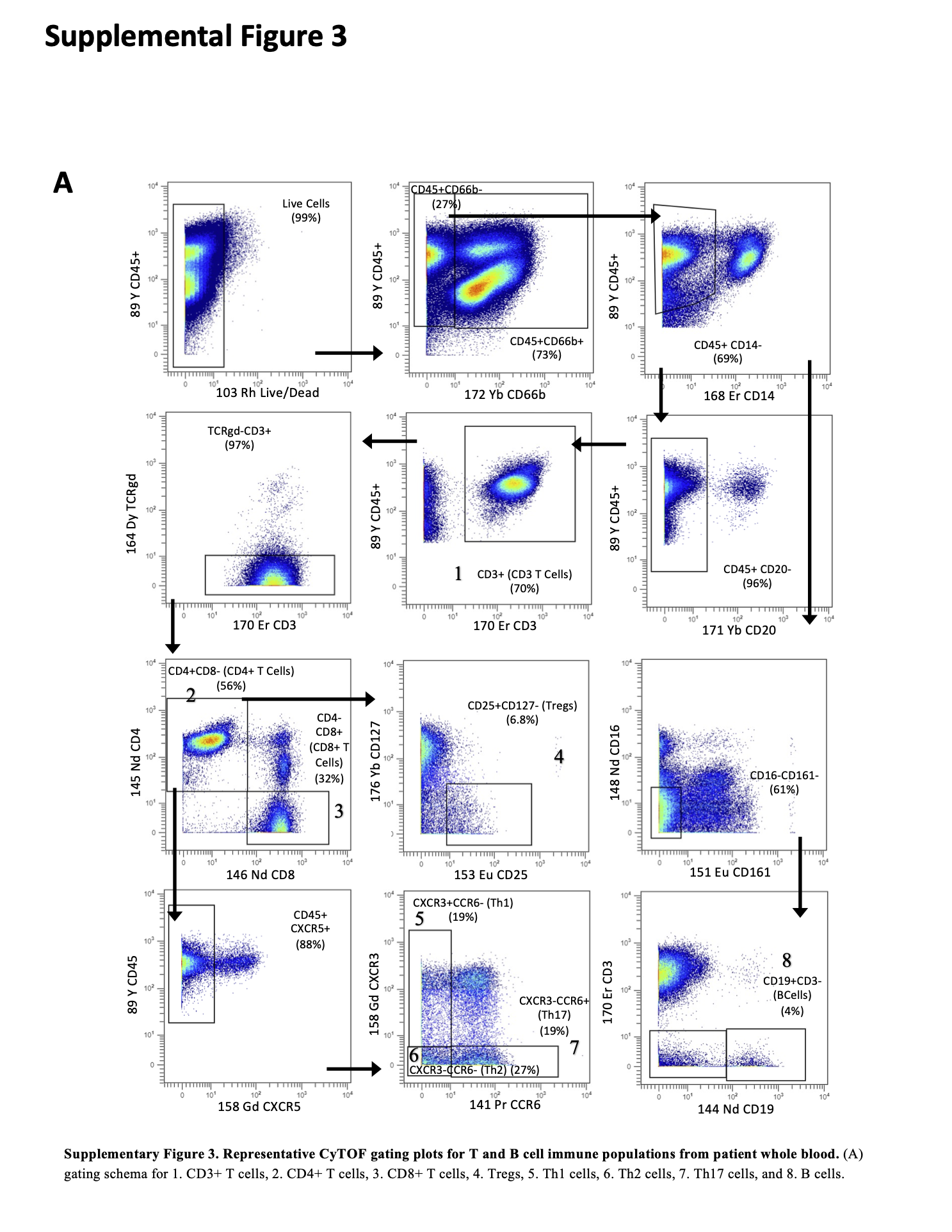

Supplement: Supplementary file 8 [file Image7.tiff]

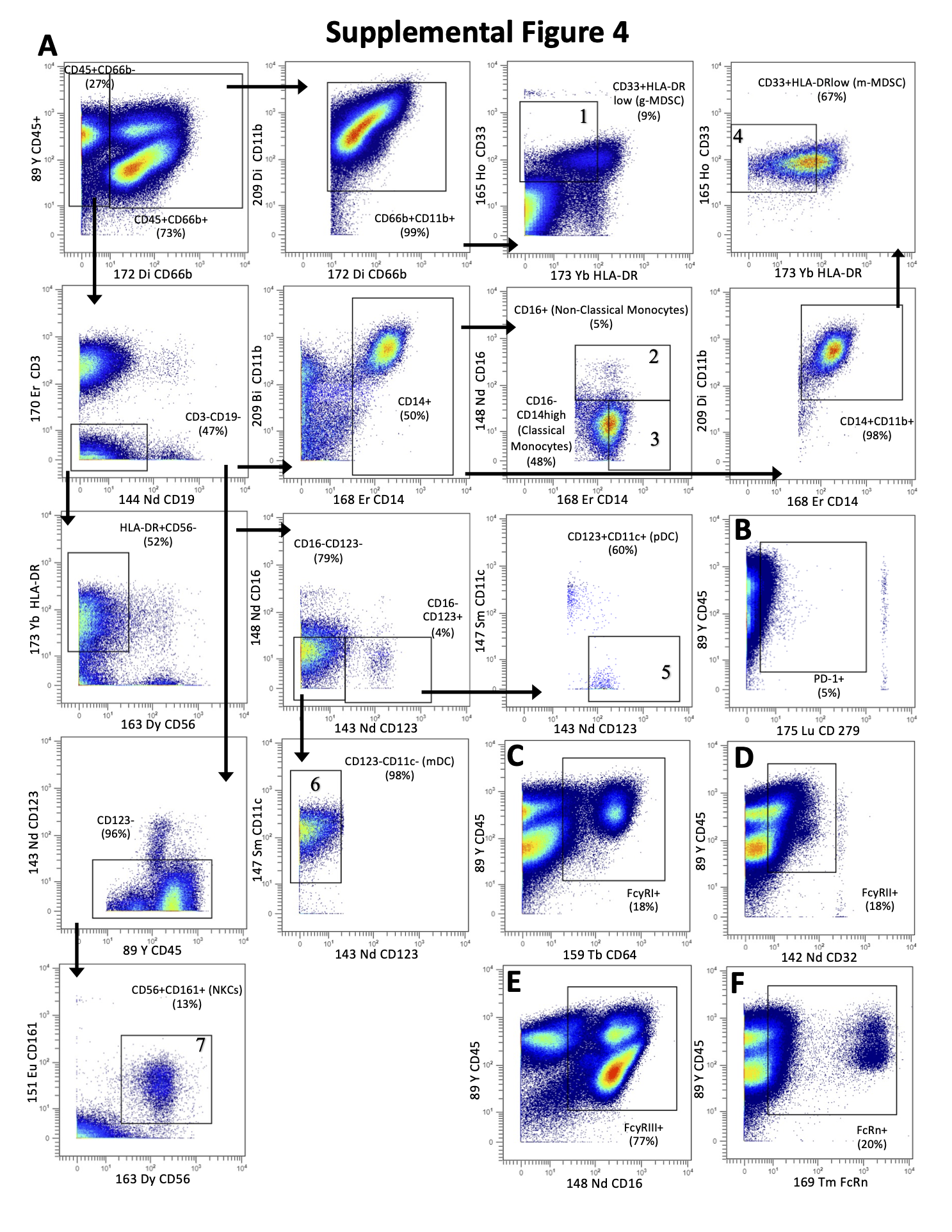

Supplement: Supplementary file 9 [file Image8.tiff]

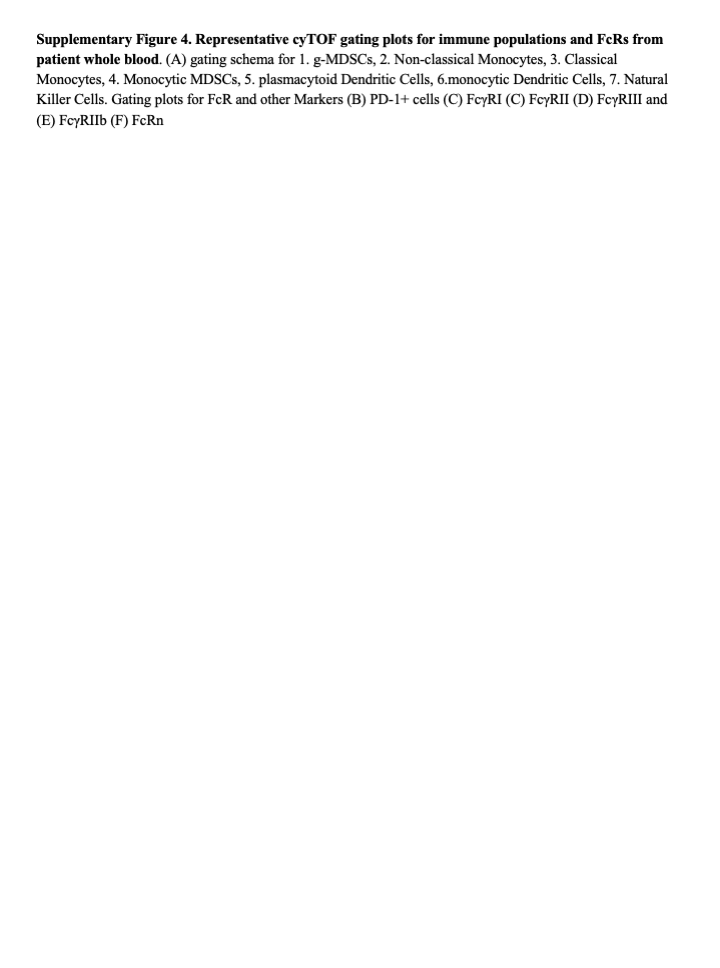

Supplement: Supplementary file 10 [file Image9.tiff]

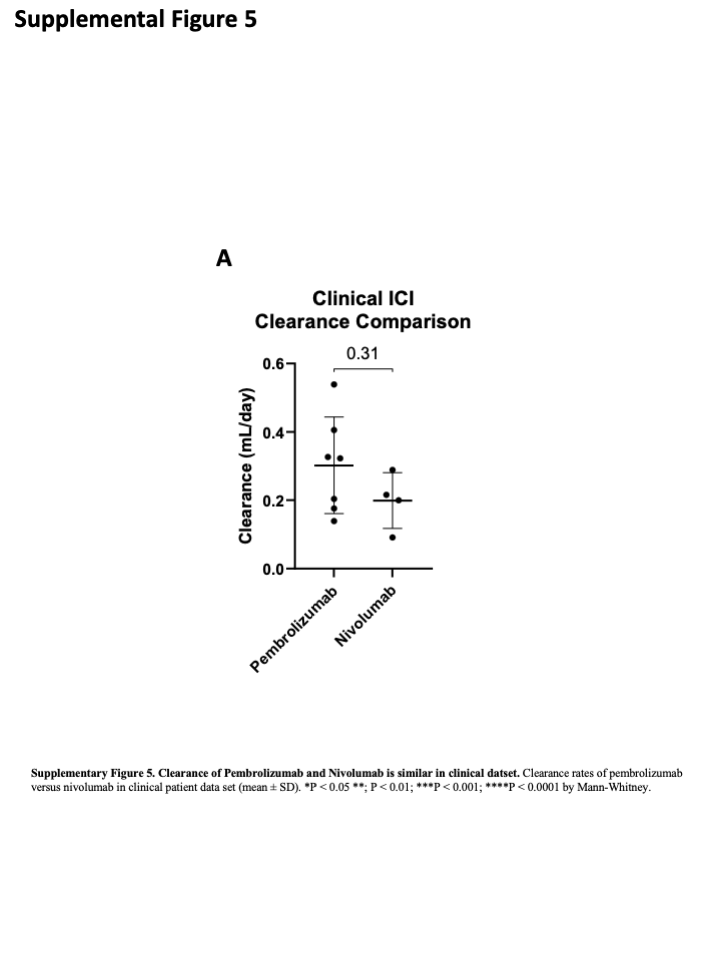

Supplement: Supplementary file 11 [file Image10.tiff]

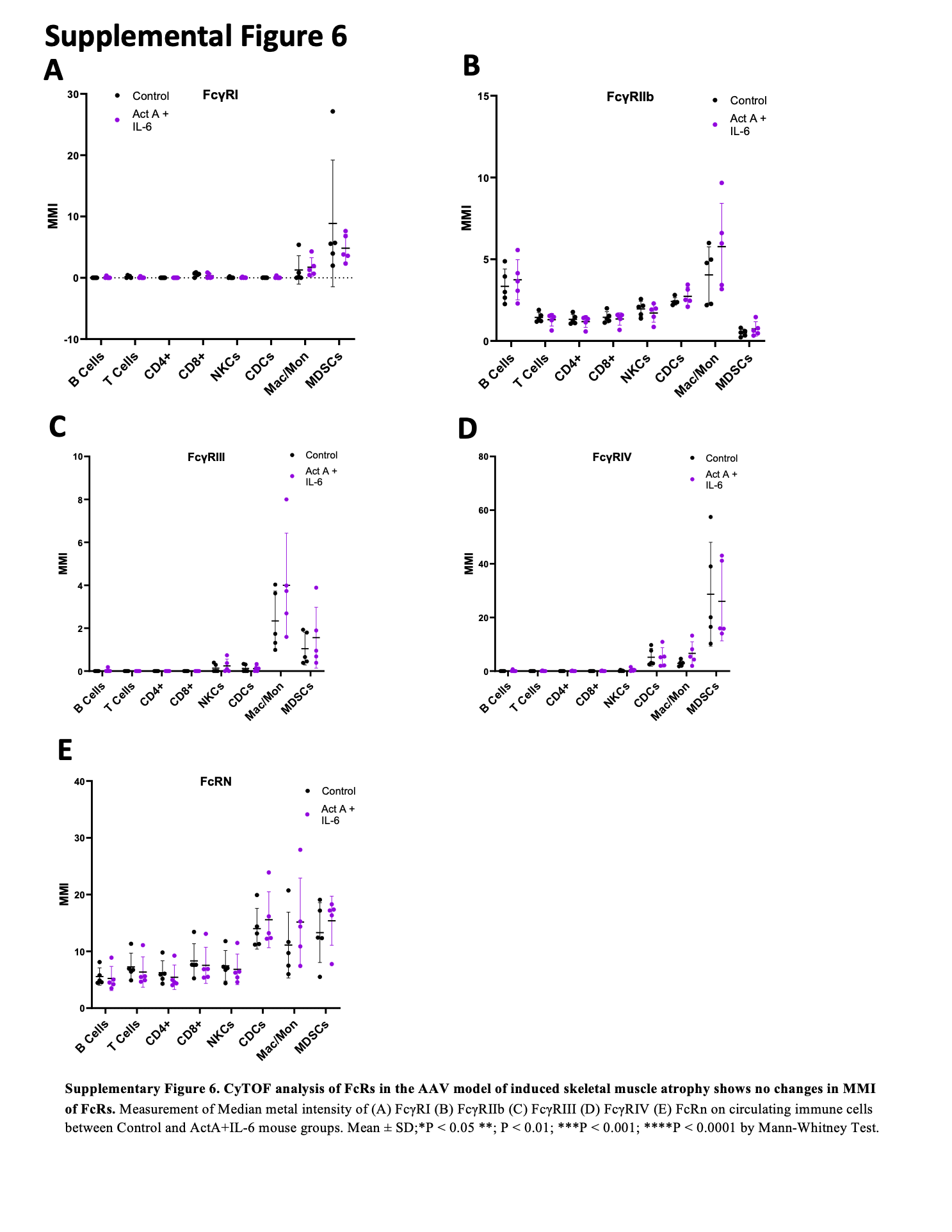

Supplement: Supplementary file 12 [file Image11.tiff]
